# The effects of saffron supplementation on cardiovascular risk factors in adults: A systematic review and dose-response meta-analysis

**DOI:** 10.3389/fnut.2022.1055517

**Published:** 2022-12-08

**Authors:** Mohammad Zamani, Mahtab Zarei, Mahlagha Nikbaf-Shandiz, Fatemeh Gholami, Amir Mehdi Hosseini, Maryam Nadery, Farideh Shiraseb, Omid Asbaghi

**Affiliations:** ^1^Department of Clinical Nutrition, School of Nutritional Sciences and Dietetics, Tehran University of Medical Sciences, Tehran, Iran; ^2^Department of Cellular and Molecular Nutrition, School of Nutritional Sciences and Dietetics, Tehran University of Medical Sciences (TUMS), Tehran, Iran; ^3^Student Research Committee, Tabriz University of Medical Sciences, Tabriz, Iran; ^4^Department of Community Nutrition, School of Nutritional Sciences and Dietetics, Tehran University of Medical Sciences (TUMS), Tehran, Iran; ^5^Faculty of Medical Sciences and Technologies, Science and Research Branch, Islamic Azad University, Tehran, Iran; ^6^Department of Dietetics and Nutrition, Robert Stempel College of Public Health & Social Work, Florida International University, Miami, FL, United States; ^7^Cancer Research Center, Shahid Beheshti University of Medical Sciences, Tehran, Iran; ^8^Student Research Committee, Shahid Beheshti University of Medical Sciences, Tehran, Iran

**Keywords:** saffron, cardiovascular risk factors, systematic review, meta-analysis, adult

## Abstract

**Introduction:**

Cardiovascular disease (CVD) is one of the leading causes of death and disability in the world and is estimated to involve more people in the next years. It is said that alternative remedies such as herbs can be used to manage the complications of this disease. For this reason, we aimed to conduct this meta-analysis to systematically assess and summarize the effects of saffron supplementation as an important herb on cardiovascular risk factors in adults.

**Methods:**

A systematic search was done in PubMed, Scopus, and Web of Science to find eligible articles up to September 2022. Randomized controlled trials (RCTs) that evaluated the effects of saffron on lipid profiles, glycemic control, blood pressure, anthropometric measures, and inflammatory markers were included. In the meta-analysis, 32 studies were taken into account (*n* = 1674).

**Results:**

Consumption of saffron significantly decreased triglyceride (TG) (WMD = −8.81 mg/dl, 95%CI: −14.33, −3.28; *P* = 0.002), total cholesterol (TC) (WMD = −6.87 mg/dl, 95%CI: −11.19, −2.56; *P* = 0.002), low density lipoprotein (LDL) (WMD = −6.71 mg/dl, 95%CI: −10.51, −2.91; *P* = 0.001), (*P* = 0.660), fasting blood glucose (FBG) level (WMD = −7.59 mg/dl, 95%CI: −11.88, −3.30; *P* = 0.001), HbA1c (WMD = −0.18%, 95%CI: −0.21, −0.07; *P* < 0.001), homeostasis model assessment-insulin resistance (HOMA-IR) (WMD = −0.49, 95%CI: −0.89, −0.09; *P* = 0.016), systolic blood pressure (SBP) (WMD = −3.42 mmHg, 95%CI: −5.80, −1.04; *P* = 0.005), tumor necrosis factor α (TNF-α) (WMD = −2.54 pg/ml, 95%CI: −4.43, −0.65; *P* = 0.008), waist circumference (WC) (WMD = −1.50 cm; 95%CI: −2.83, −0.18; *P* = 0.026), malondialdehyde (MDA) (WMD = −1.50 uM/L, 95%CI: −2.42, −0.57; *P* = 0.001), and alanine transferase (ALT) (WMD = −2.16 U/L, 95%CI: −4.10, −0.23; *P* = 0.028). Also, we observed that saffron had an increasing effect on total antioxidant capacity (TAC) (WMD = 0.07 mM/L, 95%CI: 0.01, 0.13; *P* = 0.032). There was linear regression between FBG and the duration of saffron intake. Additionally, the non-linear dose-response analysis has shown a significant association of saffron intervention with HDL (*P* = 0.049), HOMA-IR (*P* = 0.002), weight (*P* = 0.036), ALP (*P* = 0.016), FBG (*P* = 0.011), HbA1c (*P* = 0.002), and TNF-α (*P* = 0.042). A non-linear association between the length of the intervention and the level of HDL and DBP was also found.

**Discussion:**

That seems saffron could effectively improve TG, TC, LDL, FBG, HbA1c, HOMA-IR, SBP, CRP, TNF-α, WC, MDA, TAC, and ALT.

## Introduction

Cardiovascular disease (CVD) is known as one of the main causes of morbidity and mortality in societies (1). This complication which includes ischemic heart disease, stroke, heart failure, peripheral arterial disease, and other conditions (2), reduces the quality of life and life expectancy among patients and also leads to high medical care expenses on health systems and governments in different countries around the world (3, 4). Numbers show that the global prevalence of CVD almost doubled from 271 million in 1990 to 523 million in 2019 besides reaching a mortality rate from 12.1 to 18.6 million which was a third of all death globally (5). It is estimated that CVD would be the cause of more than 23 million deaths in 2030 around the world (6). Many risk factors such as gender, family history, high blood pressure, dyslipidemia, obesity, glucose abnormalities, insulin resistance, lifestyle risk factors (7, 8), and inflammation (9) are involved in the development of this disease. Accordingly, lifestyle modification especially nutritional interventions and alternative remedies like herbs can be applied to manage and treat CVD and related diseases (10, 11).

Saffron with the scientific name of “Crocus sativus Linn” (12) and bioactive compounds of crocetin, crocin, picrocrocin, and safranal (13), is a plant with medical properties (14) and is mainly cultivated in Asian and European countries (15). It has been shown that saffron could have positive impacts on hyperglycemia, insulin resistance (16), and hyperlipidemia (17) due to increasing glucose uptake and enhancing insulin sensitivity in cells (18) besides mitochondrial-β-oxidation (19). Furthermore, it is shown that this herb has anti-inflammatory and anti-oxidative benefits (18) by raising the glutathione reductase levels (20) and lowering the levels of pro-inflammatory enzymes (21). A meta-analysis conducted in 2018 on 11 RCTs showed that saffron consumption has no significant effect on improving lipid profile, fasting insulin, systolic blood pressure (SBP), and body mass index (BMI) but in subgroup analysis, a significant reduction in fasting plasma glucose levels was seen. Inflammatory factors were not examined in this study (22). Also, another meta-analysis was done in 2018 on 9 RCTs that had been conducted on diabetes and metabolic syndrome. In this study, only waist circumferences (WC), HbA1c, and fasting plasma glucose (FPG) were examined and they concluded that saffron can improve WC as well as FPG levels in sub-group analysis when intervention durations were more than 12 weeks. There was no significant effect on HbA1c levels (23). In a recent meta-analysis on 25 RCTs evaluating the effects of saffron on cardiometabolic indices in overweight and obese patients, a significant reduction in FPG was seen in participants with metabolic syndrome but there was not any considerable effect on Hb1AC, weight, and BMI (24). Besides, Rahmani’s meta-analysis containing 9 RCTs showed FPG reduction in interventions longer than 12 weeks without affecting HbA1C levels (23). Regarding lipid profile, in 2019 another meta-analysis on six RCTs showed an improvement in serum concentration of total cholesterol (TC), triglyceride (TG), and high-density lipoprotein (HDL) following supplementation with saffron but no influence on serum FPG and low-density lipoprotein (LDL) concentrations was seen (25). In addition, a meta-analysis in 2019 demonstrated the positive impact of saffron on malondialdehyde (MDA) and total antioxidant capacity (TAC) in unhealthy patients (20). Based on a 2019 article, saffron supplementation did not affect inflammatory cytokines in adults (26).

Although some studies have been done in recent years, findings show contradictory impacts of saffron and its derivates on CVD risk factors. Due to this issue and because a comprehensive meta-analysis of all the risk factors related to CVD has not been performed on new findings since then, we conducted this meta-analysis on 32 RCTs and a wide range of related variables to systematically summarize the results and evaluate the effects of saffron supplementation on cardiovascular risk factors in adults.

## Materials and methods

This systematic review and meta-analysis was performed under the preferred reporting items for systematic reviews and meta-analyses (PRISMA) guidelines (27). This study is registered at PROSPERO (CRD42022358721).

### Search strategy

To find relevant articles published up to September 2022, a systematic search was done in scientific databases including PubMed, Scopus, and Web of Science regardless of the length of studies and language. In addition, a manual search through the reference lists of relevant publications was performed to make sure we did not miss any potential studies. The PICO criteria (Participant, Intervention, Comparison/Control, Outcome) was used to search for items related to saffron supplementation and cardiovascular risk factors. (1) Participants: adults age≥18; (2) Intervention group (Saffron, Satiereal, Crocin); (3) Comparison/Control group (non-saffron supplementation), and (4) Outcome (all of the CVD risk factors that will be mentioned). The main terms and keywords we used to search the databases are as follow: (“Crocus sativus Linn” OR Safranal OR saffron OR crocin) AND (Intervention OR “Intervention Study” OR “Intervention Studies” OR “controlled trial” OR randomized OR randomized OR random OR randomly OR placebo OR “clinical trial” OR Trial OR “randomized controlled trial” OR “randomized clinical trial” OR RCT OR blinded OR “double blind” OR “double blinded” OR trial OR “clinical trial” OR trials OR “Pragmatic Clinical Trial” OR “Cross-Over Studies” OR “Cross-Over” OR “Cross-Over Study” OR parallel OR “parallel study” OR “parallel trial”).

### Study selection

Studies with the following criteria were included: (1) RCTs with either parallel or crossover design; (2) used oral supplementation of saffron; (3) investigated the effects of saffron on any of the cardiovascular risk factors and the desired variables such as triglyceride (TG), total cholesterol (TC), low-density lipoprotein (LDL), and high-density lipoprotein (HDL), fasting blood glucose (FBG), hemoglobin A1c (HbA1c), insulin, serum insulin, homeostasis model assessment-insulin resistance (HOMA-IR), systolic blood pressure (SBP), diastolic blood pressure (DBP), C-reactive protein (CRP), interleukin-6, (IL-6), tumor necrosis factor (TNF-α), total antioxidant capacity (TAC), weight, waist circumference (WC), body mass index (BMI), fat mass% (FM), aspartate transaminase (AST), alanine transaminase (ALT), malondialdehyde (MDA), alkaline phosphatase (ALP), (4) were performed on the adult population (≥ 18 years old); (5) had an intervention duration of at least four days (RCTs with two or more eligible arms were considered as separate studies); (6) provided means and standard deviations (SDs) for data, or any other effect sizes from which the calculation of mean and SD was possible; (7) human studies. Two authors (OA, MZ) independently screened the titles, abstracts, and full texts, Checked the results, and assessed the eligibility of the selected studies. Any disagreement was resolved by discussion. Exclusion criteria included animal and *in vitro* studies in addition to studies that examined the effect of another intervention along with saffron or done on children and adolescents. Moreover, studies with a non-RCT design, without a placebo group, unpublished documents, and gray literature like conference abstracts, editorial papers, and books were excluded.

### Data extraction

The following required data were extracted from eligible studies by two independent authors (OA, MZ): The first author’s name, country, publication year, type of clinical trial, participant characteristics (mean age, BMI, sex), health condition of participants, randomization, blinding, sample size, the number of participants in the intervention and control groups, the form and dose of supplemented saffron, study duration, and the desired variables. Furthermore, for both parallel and cross-over trials, means ± Standard Deviation (SD) of variables at the beginning and end of the study were collected. If this data was not available, the mean difference was calculated by subtracting the mean value at baseline from the mean value at the end of the study. If there were insufficient data in articles with pre-determined methods contact authors via email.

### Quality assessment

To assess the quality of the studies, we benefited from the Cochrane Collaboration tool (28). All the studies were checked for the probability of bias. This included randomized sequence generation, allocation concealment, blindness (participants, staff, and outcome assessment), incomplete outcome data, selective outcome reporting, and other biases. Based on the recommendations of the Cochrane Handbook, three groups of high risk of bias, low risk of bias, and uncertain risk of bias were created. The quality of studies in which the number of high-risk biases was more than 2 was considered as bad and in the same way, those having 2 or less than 2 high-risk biases were considered fair and good, respectively. The quality of the work was checked by two authors (OA, MZ) and in case of any disagreement, the problem was resolved by discussion and consulting.

### Statistical analysis

All statistical analyzes of eligible studies were performed using Stata software version 11.0 (Stata Corp, College Station, TX). All tests were two-tailed, and *p* < 0.05 were considered statistically significant. The pooled weighted mean difference (WMD) was calculated by a random-effects model to consider the existing heterogeneity (29) and also the Interstudy heterogeneity was performed using I-square (I^2^) test (30), with values greater than 40% indicating strong heterogeneity (31). The mean differences of required variables in both intervention and control groups at the beginning and end of the study were calculated and also the SD of these mean differences was computed using the following formula: SD = square root [(SD at baseline)^2^ + (SD at the end of study)^2^ − (2r × SD at baseline × SD at the end of study)] (32). All standard errors (SEs), 95 percent confidence intervals (CIs), and interquartile ranges (IQRs) to SDs which had been reported in studies, converted to SD using a method introduced by Hozo et al. and this formula: SD = SE × √n (n = the number of individuals in each group) (33). We applied a correlation coefficient of 0.8 for r (28). To define the source of heterogeneity, a subgroup analysis was done. Subgroups were selected based on the required minimum number of studies according to the established criteria, where there should be at least 6 to 10 studies for continuous and a minimum of 4 studies for categorical subgroup variables (34, 35). The analysis of baseline TG (<150 mg/dl, ≥150 mg/dl), TC (<200 mg/dl, ≥200 mg/dl), LDL (<100 mg/dl, ≥100 mg/dl), HDL (<40 mg/dl, ≥40 mg/dl), FBG (<100 mg/dl, ≥100 mg/dl), SBP (<120 mmHg, ≥120 mmHg), DBP (<80 mmHg, ≥80 mmHg), Intervention duration (≤12 weeks, >12 weeks), and dosage of saffron (<100 mg/day, ≥100 mg/day) were based on the median values of the included studies. Other subgroup analyses were performed according to health status (diabetic, non-diabetic), and baseline BMI [normal (18.5–24.9 kg/m^2^), overweight (25–29.9 kg/m^2^), and obese (≥30 kg/m^2^)].

The potential publication bias was reviewed by a funnel plot test (36, 37). Sensitivity analyses were conducted to explore the impact of each study on the pooled effect size. We used the trim-and-fill method to detect and adjust the publication bias’s impact (38). Meta-regression was performed to evaluate the potential effects of saffron (mg/d) dosage and duration on the variables. Furthermore, we used non-linear regression for dose-response analysis between saffron supplementation and our variables.

### Certainty assessment

The overall quality of evidence in all studies was assessed and summarized using the GRADE (Grading of Recommendations Assessment, Development, and Evaluation) approach (39).

## Result

### The flow of study selection

**FIGURE 1:**
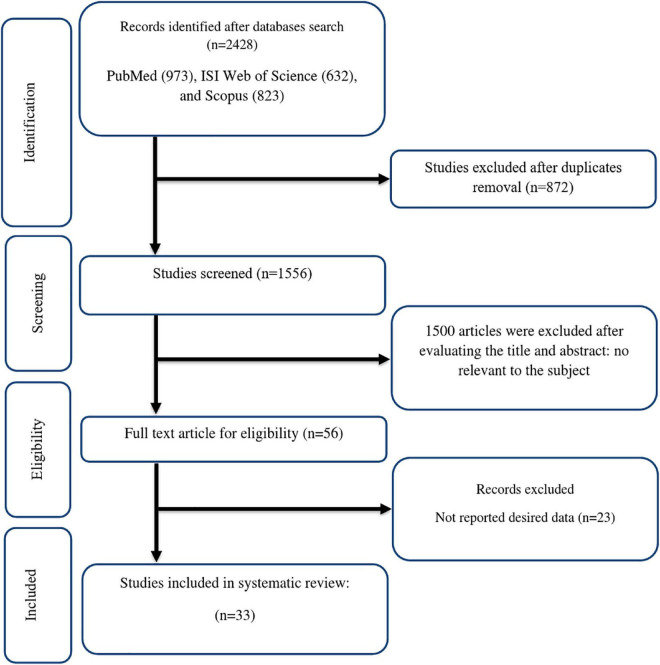
Flow chart of study selection for inclusion trials in the systematic review.

Initially, 2428 potentially eligible records were found in the literature using an electronic search [PubMed (973), ISI Web of Science (632), and Scopus (823)]. After duplicates were eliminated (*n* = 873) and title/abstract screening, 1500 articles were excluded because they had no relevance to the topic. As a result, 55 full-text papers were collected for a thorough evaluation. 23 of these studies had papers with no useful data (Figure 1). Finally, 32 trials (15, 17, 40–69), were considered eligible for the systematic review. The meta-analysis was conducted on 24 effect sizes for TG (15, 17, 40, 42, 44, 45, 47, 49, 51–55, 57, 58, 60, 61, 63, 67), 23 for TC (15, 17, 40, 42, 44, 45, 47, 49, 51–55, 57, 58, 60, 61, 63), 23 for LDL (15, 17, 40, 44, 45, 47, 49, 51–55, 57, 58, 60, 61, 63, 67), 23 for HDL (15, 17, 40, 44, 45, 47, 49, 51–55, 57, 58, 60, 61, 63, 67), 22 for FBG (15, 17, 42, 44, 45, 47, 49, 51–54, 57, 58, 60–62, 64, 66, 67), 7 for insulin (15, 44, 45, 57, 58, 64), 12 for HbA1c (15, 17, 44, 45, 54, 57, 60–62, 64), 7 for HOMA-IR (15, 45, 57, 58, 61, 64), 11 for SBP (40, 45, 48, 52, 53, 57, 63, 64, 66), 9 for DBP (40, 48, 52, 53, 57, 63, 64, 66), 10 for CRP (44, 46, 52, 57, 58, 62, 65, 68), 3 for IL-6 (53, 62, 66), 7 for TNF-α (53, 57, 59, 62, 65, 66, 68), 13 for weight (41, 44, 48–50, 52, 55, 57, 58, 60, 68, 69), 12 for BMI (44, 48–50, 52, 57, 58, 60, 63, 68, 69), 14 for WC (41, 44, 45, 48, 49, 52, 53, 55, 57, 60, 68, 69), 5 for FM% (41, 49, 57, 68), 7 for MDA (57–59, 62, 65, 68, 69), 7 for TAC (57–59, 62, 65, 68, 69), 11 for ALT (15, 42, 46, 54, 57, 61, 67, 68), 11 for AST (15, 42, 46, 54, 57, 61, 67, 68), and 5 for ALP (42, 46, 57, 61).

### Study characteristics

Table 1 lists the characteristics of the trials that included a total of 1674 participants who were enrolled in the studies, 842 of them were assigned to the intervention group and 832 to the control group. All publications that were included in the present systematic review were randomized controlled clinical trials in design, and a parallel research design was used in all studies (15, 17, 40–69). All of these investigations were conducted in France (41) and Iran (15, 17, 40, 42–69), and were published between 2008 and 2021. The participants’ average ages ranged from 27 to 57.95, while their average baseline BMIs ranged from 23.84 to 31.02 kg/m^2^. The follow-up period ranged from 1 to 12 weeks. Daily supplementation dosage of saffron varied between 5 (54) and 1000 (44, 48) mg/day in these studies. In two studies (40, 54), data were reported for two different doses, hence four effect sizes were calculated. Six effect sizes were estimated as a result of the three studies (45, 46, 49) data on two varieties of saffron being provided. Only one (46) of the included studies had a male-only population, two (41, 65) had a female-only population, and the remaining trials (15, 17, 40, 42–45, 47–64, 66–69) involved mixed-gender populations.

**TABLE 1:** Characteristics of included studies in the meta-analysis.

References	Country	Study design	Participant	Sample size and sex	Sample size		Trial duration (Week)	Means age		Means BMI		Intervention			Adverse events
					IG	CG		IG	CG	IG	CG	Type of intervention	Intervention (mg/d)	Control group	
Modaghegh et al. (40)	Iran	Parallel, R, PC, DB	healthy volunteers	M/F: 20	10	10	1	27 ± 6.5	28.7 ± 6.22	NR	NR	Saffron	200	Placebo	No major adverse events
Modaghegh et al. (40)	Iran	Parallel, R, PC, DB	healthy volunteers	M/F: 20	10	10	1	28.7 ± 5.5	28.7 ± 6.22	NR	NR	Saffron	400	Placebo	No major adverse events
Gout et al. (41)	France	Parallel, R, PC, DB	mildly overweight healthy women	F: 60	31	29	8	36.2 ± 5.5	35.9 ± 5.4	26.7 ± 1.2	26.9 ± 1.1	Satiereal	176.5	Placebo	Mild side effects
Mansoori et al. (42)	Iran	Parallel, R, PC, DB	patients with major depressive disorder	M/F: 20	10	10	4	35.3 ± 5.81	42.4 ± 8.44	NR	NR	Saffron	30	Placebo	Dry mouth (n = 3), Restlessness (n = 2), Anxiety (n = 2), Daily drowsiness (n = 1), Morning drowsiness
Mohamadpour et al. (43)	Iran	Parallel, R, PC, DB	Healthy Volunteers	M/F: 44	22	22	4	31.1 ± 13	31.1 ± 13	24.9 ± 7.1	24.9 ± 7.1	Crocin	20	Placebo	No major adverse events
Fadai et al. (45)	Iran	Parallel, R, PC, TB	Patients with Schizophrenia	M/F: 44	22	22	12	48.1 ± 7.7	48.1 ± 6.1	NR	NR	Crocin	30	Placebo	No serious adverse effects
Fadai et al. (45)	Iran	Parallel, R, PC, TB	Patients with Schizophrenia	M/F: 44	22	22	12	49.3 ± 7.1	48.1 ± 6.1	NR	NR	Saffron Aqueous Extract	30	Placebo	No serious adverse effects
Azimi et al. (44)	Iran	Parallel, R, PC, SB	Type 2 diabetes	M/F: 81	42	39	8	57.02 ± 6.5	53.64 ± 7.9	28.86 ± 1.6	28.4 ± 1.3	Saffron	1000	Control group	No adverse events
Mousavi et al. (46)	Iran	Parallel, R, PC, DB	patients with schizophrenia	M: 44	22	22	12	48.1 ± 7.7	48.1 ± 6.1	NR	NR	Crocin	30	Placebo	No serious adverse effects
Mousavi et al. (46)	Iran	Parallel, R, PC, DB	patients with schizophrenia	M: 44	22	22	12	49.3 ± 7.1	48.1 ± 6.1	NR	NR	Saffron Aqueous Extract	30	Placebo	No serious adverse effects
Nikbakht-Jam et al. (47)	Iran	Parallel, R, PC, DB	Subjects with Metabolic Syndrome	M/F: 60	30	30	8	38.97 ± 13.33	43.46 ± 12.77	NR	NR	Crocin	30	Control group	NR
Azimi et al. (48)	Iran	Parallel, R, PC, SB	Type 2 diabetes	M/F: 81	42	39	8	57.02 ± 6.8	53.64 ± 7.8	28.86 ± 1.5	28.4 ± 1.3	Saffron	1000	Control group	No adverse effects
Javandoost et al. (51)	Iran	Parallel, R, PC, DB	subjects with metabolic syndrome	M/F: 44	22	22	8	38.8 ± 12	40.45 ± 11.2	NR	NR	Crocin	30	Placebo	NR
Abedimanesh et al. (49)	Iran	Parallel, R, PC, DB	patients with coronary artery disease	M/F: 50	25	25	8	53.36 ± 5.94	56.32 ± 5.91	27.92 ± 2.57	28.05 ± 2.89	Crocin	30	Placebo	No serious adverse effects
Abedimanesh et al. (49)	Iran	Parallel, R, PC, DB	patients with coronary artery disease	M/F: 50	25	25	8	56.04 ± 7.55	56.32 ± 5.91	28.64 ± 2.23	28.05 ± 2.89	Saffron Aqueous Extract	30	Placebo	No serious adverse effects
Kermani et al. (52)	Iran	Parallel, R, PC, DB	Subjects with Metabolic Syndrome	M/F: 44	22	22	12	43.64 ± 11.17	42.59 ± 8.44	31.02 ± 5.45	30.48 ± 6.26	Saffron	100	Placebo	NR
Kermani et al. (53)	Iran	Parallel, R, PC, DB	Metabolic Syndrome	M/F: 48	24	24	6	53.8 ± 9.2	50.9 ± 8.8	29.9 ± 3.9	29.8 ± 5.3	Saffron	100	Placebo	No adverse effects
Jafarnia et al. (50)	Iran	Parallel, R, PC, DB	Mild to Moderate Generalized Anxiety Disorder	M/F: 40	20	20	6	29.65 ± 8.45	32.4 ± 6.74	26.33 ± 5.12	25.49 ± 5.9	Saffron	450	Placebo	Constipation (n = 1), polydipsia (n = 1), headache (n = 2)
Milajerdi et al. (17)	Iran	Parallel, R, PC, TB	Type 2 diabetes	M/F: 54	27	27	8	54.57 ± 6.96	55.42 ± 7.58	23.84 ± 11.89	28.3 ± 3.24	Saffron	30	Placebo	Headache
Sepahi et al. (54)	Iran	Parallel, R, PC, DB	patients with refractory diabetic maculopathy	M/F: 68	34	34	12	54.31 ± 6.6	57.17 ± 2.9	NR	NR	Crocin	5	Placebo	Increased appetite, feet swelling, stomach ache, subconjunctival-hemorrhage, swelling, redness, and burning of the eyes
Sepahi et al. (54)	Iran	Parallel, R, PC, DB	patients with refractory diabetic maculopathy	M/F: 67	33	34	12	56.09 ± 4.3	57.17 ± 2.9	NR	NR	Crocin	15	Placebo	Increased appetite, feet swelling, stomach ache, subconjunctival-hemorrhage, swelling, redness, and burning of the eyes
Zilaee et al. (55)	Iran	Parallel, R, PC, DB	patients with metabolic syndrome	M/F: 76	38	38	12	42.19 ± 11.52	43.6 ± 9.05	NR	NR	Saffron	100	Placebo	NR
Moravej Aleali et al. (61)	Iran	Parallel, R, PC, DB	Type 2 diabetes	M/F: 64	32	32	12	53.5 ± 9.9	52.4 ± 13	28.8 ± 4	27.5 ± 4.2	Saffron	15	Placebo	NR
Ghaderi et al. (58)	Iran	Parallel, R, PC, DB	patients under methadone maintenance treatment	M/F: 53	26	27	8	44.5 ± 9.4	45.6 ± 9.9	24.5 ± 4.4	25.2 ± 4.2	Crocin	15	Placebo	No adverse effects
Ebrahimi et al. (56)	Iran	Parallel, R, PC, DB	Type 2 diabetes	M/F: 80	40	40	12	55.2 ± 7.3	53 ± 10.6	28.7 ± 4.15	29.91 ± 3.91	Saffron	100	Placebo	NR
Karimi-Nazari et al. (60)	Iran	Parallel, R, PC, DB	overweight/ obese prediabetic	M/F: 75	36	39	8	57.95 ± 8.12	57.9 ± 8.7	29.35 ± 1.5	28.78 ± 2.02	Saffron	15	Placebo	No adverse effects
Shahbazian et al. (62)	Iran	Parallel, R, PC, DB	Type 2 diabetes	M/F: 64	32	32	12	53.5 ± 9.9	52.4 ± 13	28.8 ± 4	27.5 ± 4.2	Saffron	15	Placebo	NR
Zilaee et al. (63)	Iran	Parallel, R, PC, DB	patients with mild and moderate persistent allergic asthma	M/F: 76	38	38	8	41.27 ± 9.77	40.77 ± 10.07	26.84 ± 1.9	26.84 ± 2.34	Saffron	100	Placebo	No serious adverse effects
Ghiasian et al. (59)	Iran	Parallel, R, PC, DB	multiple sclerosis patients	M/F: 40	20	20	4	29 ± 4.99	31.47 ± 5.31	NR	NR	Crocin	30	Placebo	NR
Ebrahimi et al. (57)	Iran	Parallel, R, PC, DB	Type 2 diabetes	M/F: 80	40	40	12	55.2 ± 7.3	53 ± 10.6	29.3 ± 4.9	30.5 ± 4.7	Saffron	100	Placebo	NR
Behrouz et al. (64)	Iran	Parallel, R, PC, DB	Type 2 diabetes	M/F: 50	25	25	12	57.08 ± 7.41	59.86 ± 9.46	30.64 ± 4.79	30.85 ± 3.19	Crocin	30	Placebo	No serious adverse effects
Mobasseri et al. (66)	Iran	Parallel, R, PC, DB	Type 2 diabetes	M/F: 57	30	27	8	50.57 ± 9.88	51.63 ± 11.3	30.96 ± 4.23	31.02 ± 4.69	Saffron	100	Placebo	NR
Parsi et al. (67)	Iran	Parallel, R, PC, DB	patients with non-alcoholic fatty liver disease	M/F: 60	30	30	8	33.08 ± 2.8	36.1 ± 5.47	29.84 ± 3.37	30.25 ± 3.31	Crocin	15	Placebo	NR
Hamidi et al. (65)	Iran	Parallel, R, PC, DB	patients with active rheumatoid arthritis	F: 66	33	33	12	51.55 ± 8.26	51.8 ± 9.62	28.17 ± 3.74	28.39 ± 3.7	Saffron	100	Placebo	Stomach pain.
Kavianipour et al. (68)	Iran	Parallel, R, PC, DB	patients with non-alcoholic fatty liver disease	M/F: 76	38	38	12	43.42 ± 10.62	42.05 ± 8.27	28.85 ± 5.45	29.6 ± 4.4	Saffron	100	Placebo	No adverse effects
Tajaddini et al. (15)	Iran	Parallel, R, PC, DB	Type 2 diabetes	M/F: 70	35	35	8	50.5 ± 9.8	51.8 ± 10.9	30 ± 4.2	31.2 ± 4.6	Saffron	100	Placebo	No adverse effects
Tahvilian et al. (69)	Iran	Parallel, R, PC, DB	ulcerative colitis patients	M/F: 75	40	35	8	40.55 ± 12.71	40.97 ± 11.34	26.95 ± 10.68	24.8 ± 3.46		100	Placebo	NR

IG, intervention group; CG, control group; DB, double-blinded; SB, single-blinded; PC, placebo-controlled; CO, controlled; RA, randomized; NR, not reported; F, female; M, male; NR, not reported.

Age: mean age of participants; BMI: mean of body mass index.

**FIGURE 2:**
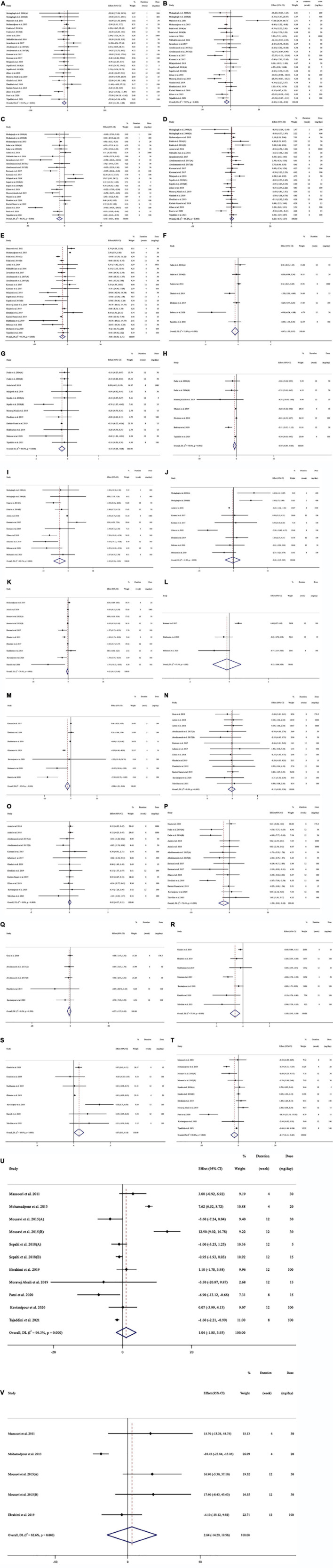
Forest plot detailing weighted mean difference and 95% confidence intervals (CIs) for the effect of saffron consumption on **(A)** TG (mg/dl); **(B)** TC (mg/dl); **(C)** LDL (mg/dl); **(D)** HDL (mg/dl); **(E)** FBG (mg/dl); **(F)** Insulin (miu/ml); **(G)** HbA1c (%); **(H)** HOMA-IR; **(I)** SBP (mmHg); **(J)** DBP (mmHg); **(K)** CRP (mg/l); **(L)**; IL-6 (pg/ml); **(M)** TNF-α (pg/ml); **(N)** weight (kg); **(O)** BMI (kg/m^2^); **(P)** WC (cm); **(Q)** FM (%); **(R)** MDA (uM/L); **(S)** TAC (mM/L); **(T)** ALT (U/L); **(U)** AST (U/L) and **(V)** ALP (U/L). TG, triglyceride; TC, total cholesterol; LDL, low-density lipoprotein; HDL, high-density lipoprotein; FBG, fasting blood glucose; HOMA-IR, homeostasis model assessment for insulin resistance; HbA1C, hemoglobin A1C; CRP, C-reactive protein; IL-6, interleukin 6; TNF-α, tumor necrosis factor; TAC, total antioxidant capacity; BMI, body mass index; WC, waist circumference; FM, fat mass; ALT, alanine transaminase; AST, aspartate transaminase; ALP, alkaline phosphatase; MDA, malondialdehyde*;* SBP, systolic blood pressure; DBP, diastolic blood pressure; CI, confidence interval; WMD, weighted mean difference.

Subjects with a variety of health conditions were all included in the study: type 2 diabetes patients (15, 17, 44, 48, 56, 57, 61, 62, 64, 66), patients with schizophrenia (45, 46), patients with major depressive disorder (42), patients with coronary artery disease (49), patients with refractory diabetic maculopathy (54), subjects with mild to moderate generalized anxiety disorder (50), individuals with metabolic syndrome (47, 51–53, 55), healthy subjects (40, 43), mildly overweight healthy women (41), patients under methadone maintenance treatment (58), overweight/obese prediabetic patients (60), patients with mild and moderate persistent allergic asthma (63), multiple sclerosis patients (59), patients with non-alcoholic fatty liver disease (67, 68), patients with active rheumatoid arthritis (65), and ulcerative colitis patients (69). All research was done in English. Figure 2A (TG), 2B (TC), 2C (LDL), 2D (HDL), 2E (FBG), 2F (insulin), 2G (HbA1c), 2H (HOMA-IR), 2I (SBP), 2J (DBP), 2K (CRP), 2L (IL-6), 2M (TNF-α), 2N (weight), 2O (BMI), 2P (WC), 2Q (FM%), 2R (MDA), 2S (TAC), 2T (ALT), 2U (AST), and 2V (ALP) depict the WMD and 95% CI for outcomes forest plots.

### Adverse events

Most studies did not report specific side effects, but some side effects such as dry mouth, restlessness, anxiety, daily drowsiness, morning drowsiness (42), constipation, polydipsia, headache (17, 50), increased appetite, feet swelling, stomach ache, subconjunctival-hemorrhage, swelling, redness, and burning of the eyes (54), stomach pain (65), were reported in some studies.

### Quality assessment

Out of the 32 studies examined for this review, 11 trials (15, 17, 50, 56, 57, 60–63, 68, 69) were rated as having good quality, 12 trials (40, 41, 45, 47, 49, 51, 54, 58, 64–67) as having medium quality, and 9 trials (42–44, 46, 48, 52, 53, 59, 63) as having low quality. The details of the risk of bias in studies according to the domains used by the Cochrane collaboration’s tool are provided in Table 2.

**TABLE 2:** Quality assessment (A summary of the risk of bias according to Cochrane criteria).

References	Random sequence generation	Allocation concealment	Selective reporting	Other sources of bias	Blinding (participants and personnel)	Blinding (outcome assessment)	Incomplete outcome data	General risk of bias	Quality
Modaghegh et al. (40)	L	H	H	L	L	U	L	M	Fair
Gout et al. (41)	L	L	H	H	L	U	L	M	Fair
Mansoori et al. (42)	L	H	H	H	L	U	L	H	Bad
Mohamadpour et al. (43)	L	L	H	H	L	U	H	H	Bad
Fadai et al. (45)	L	L	H	H	L	L	L	M	Fair
Azimi et al. (44)	L	H	H	L	H	H	L	H	Bad
Mousavi et al. (46)	L	H	H	H	L	U	L	H	Bad
Nikbakht-Jam et al. (47)	L	L	H	H	L	U	L	M	Fair
Azimi et al. (48)	L	H	H	H	H	H	L	H	Bad
Javandoost et al. (51)	L	L	H	H	L	U	L	M	Fair
Abedimanesh et al. (49)	L	H	H	L	L	U	L	M	Fair
Kermani et al. (52)	L	H	H	H	L	U	L	H	Bad
Kermani et al. (53)	L	H	H	H	L	U	L	H	Bad
Jafarnia et al. (50)	L	L	H	L	L	U	L	L	Good
Milajerdi et al. (17)	L	L	H	L	L	L	L	L	Good
Sepahi et al. (54)	L	L	H	H	L	U	L	M	Fair
Zilaee et al. (55)	L	H	H	H	L	U	L	H	Bad
Moravej Aleali et al. (61)	L	L	H	L	L	U	L	L	Good
Ghaderi et al. (58)	L	L	H	H	L	U	L	M	Fair
Ebrahimi et al. (56)	L	L	H	L	L	U	L	L	Good
Karimi-Nazari et al. (60)	L	L	H	L	L	U	L	L	Good
Shahbazian et al. (62)	L	L	H	L	L	U	L	L	Good
Zilaee et al. (63)	L	L	H	L	L	U	L	L	Good
Ghiasian et al. (59)	L	H	H	H	L	U	L	H	Bad
Ebrahimi et al. (57)	L	L	H	L	L	U	L	L	Good
Behrouz et al. (64)	L	L	H	H	L	U	L	M	Fair
Mobasseri et al. (66)	L	L	H	H	L	U	L	M	Fair
Parsi et al. (67)	L	L	H	H	L	U	L	M	Fair
Hamidi et al. (65)	L	L	H	H	L	U	L	M	Fair
Kavianipour et al. (68)	L	L	H	L	L	U	L	L	Good
Tajaddini et al. (15)	L	L	H	L	L	U	L	L	Good
Tahvilian et al. (69)	L	L	H	L	L	U	L	L	Good

H, high risk of bias; L, low risk of bias; U, unclear risk of bias.

The Cochrane collaboration tool was used to assess the quality of studies.

Bad > 2 high risks; Good < 2 high risk; Fair = 2 high risk.

### Meta-analysis

#### Effect of saffron consumption on lipid profiles and subgroup analysis

In total, we pooled 24 effect sizes from 18 studies, with 1312 participants [intervention group (IG) = 655, control group (CG) = 657], to estimate the effect of saffron on plasma TG (15, 17, 40, 42, 44, 45, 47, 49, 51–55, 57, 58, 60, 61, 63, 67), and 23 effect sizes from 18 studies, for TC (15, 17, 40, 42, 44, 45, 47, 49, 51–55, 57, 58, 60, 61, 63) with 1208 participants (IG = 603, CG = 605), LDL (15, 17, 40, 44, 45, 47, 49, 51–55, 57, 58, 60, 61, 63, 67) with 1292 participants (IG = 645, CG = 647), and HDL (15, 17, 40, 44, 45, 47, 49, 51–55, 57, 58, 60, 61, 63, 67) levels with 1292 participants (IG = 645, CG = 647) (Table 3). According to the overall result of the meta-analysis, saffron significantly decreased serum TG (WMD = −8.81 mg/dl, 95%CI: −14.33, −3.28; *P* = 0.002; *I*^2^ = 55.1%, *P* = 0.001; Figure 2A), TC (WMD = −6.87 mg/dl, 95%CI: −11.19, −2.56; P = 0.002; I^2^ = 72.5%, P < 0.001; Figure 2B), and LDL (WMD = −6.71 mg/dl, 95%CI: −10.51, −2.91; P = 0.001; I^2^ = 81.3%, P < 0.001; Figure 2C). However, saffron on HDL showed no significant effect (WMD = 0.21 mg/dl, 95%CI: −0.73, 1.16; P = 0.660; I^2^ = 66.2%, P < 0.001; Figure 2D).

**TABLE 3:** Subgroup analyses of saffron on CVD risk factors in adults.

	NO	WMD (95%CI)	*P*-value	Heterogeneity		
				P heterogeneity	I^2^	P between sub-groups
**Subgroup analyses of saffron on serum TG (mg/dl)**						
Overall effect	24	−8.81 (−14.33, −3.28)	**0.002**	0.001	55.1%	
**Baseline TG (mg/dl)**						
<150	11	−4.65 (−8.88, −0.43)	**0.031**	0.430	1.2%	0.405
≥150	12	−9.95 (−21.67, 1.77)	0.096	<0.001	70.6%	
**Trial duration (week)**						
<12	16	−11.18 (−18.53, −3.84)	**0.003**	<0.001	67.7%	0.253
≥12	8	−5.04 (−12.60, 2.50)	0.190	0.686	0.0%	
**Intervention dose (mg/day)**						
<100	15	−7.80 (−14.44, −1.16)	**0.021**	0.001	60.6%	0.553
≥100	9	−11.29 (−20.69, −1.89)	**0.019**	0.178	30.0%	
**Baseline BMI (kg/m^2^)**						
Normal (18.5−24.9)	3	−8.92 (−19.09, 1.24)	0.085	0.128	51.4%	0.679
Overweight (25−29.9)	9	−11.76 (−24.49, 0.96)	0.070	<0.001	77.9%	
Obese (>30)	2	17.93 (−35.31, −0.55)	**0.043**	0.482	0.0%	
**Health status**						
Diabetic	7	−5.08 (−12.80, 2.64)	0.197	0.403	3.0%	0.295
Non-diabetic	17	−10.66 (−17.70, −3.62)	**0.003**	<0.001	64.4%	
**Intervention**						
Saffron	13	−8.96 (−16.01, −1.93)	**0.013**	0.069	39.7%	0.882
Crocin	9	−7.94 (−19.55, 3.67)	0.180	<0.001	72.9%	
**Subgroup analyses of saffron on serum TC (mg/dl)**						
Overall effect	23	−6.87 (−11.19, −2.56)	**0.002**	<0.001	72.5%	
**Baseline TC (mg/dl)**						
<200	18	−7.39 (−13.16, −1.62)	**0.012**	<0.001	77.4%	0.223
≥200	4	−1.54 (−8.96, 5.87)	0.683	0.520	0.0%	
**Trial duration (week)**						
<12	15	−4.44 (−9.45, 0.56)	0.082	<0.001	69.0%	0.180
≥12	8	−11.21 (−19.74, −2.69)	**0.010**	<0.001	76.0%	
**Intervention dose (mg/day)**						
<100	14	−4.52 (−9.96, 0.92)	0.104	<0.001	74.9%	0.181
≥100	9	−10.76 (−18.12, −3.41)	**0.004**	0.002	66.5%	
**Baseline BMI (kg/m^2^)**						
Normal (18.5−24.9)	3	−7.43 (10.52, −4.34)	**<0.001**	0.371	0.0%	
Overweight (25−29.9)	8	−6.65 (−13.77, 0.46)	0.067	<0.001	73.2%	0.236
Obese (>30)	2	−19.59 (−33.56, −5.61)	**0.006**	0.094	64.4%	
**Health status**						
Diabetic	7	−5.05 (−13.54, 3.43)	0.243	0.001	74.1%	0.594
Non-diabetic	16	−7.77 (−13.03, −2.50)	**0.004**	<0.001	73.4%	
**Intervention**						
Saffron	13	−6.88 (−14.66, 0.90)	0.083	<0.001	83.8%	0.947
Crocin	8	−7.15 (−9.98, −4.33)	**<0.001**	0.716	0.0%	
**Subgroup analyses of saffron on serum LDL (mg/dl)**						
Overall effect	23	−6.71 (−10.51, −2.91)	**0.001**	<0.001	81.3%	
**Baseline LDL (mg/dl)**						
<100	7	−4.49 (−11.88, 2.88)	0.233	0.002	71.6%	0.466
≥100	15	−8.10 (−14.38, −1.82)	**0.011**	<0.001	85.4%	
**Trial duration (week)**						
<12	15	−6.68 (−11.83, −1.52)	**0.011**	<0.001	85.7%	0.944
≥12	8	−6.94 (−12.31, −1.58)	**0.011**	0.014	60.4%	
**Intervention dose (mg/day)**						
<100	14	−5.10 (−5.10, −1.23)	**0.010**	<0.001	71.5%	0.508
≥100	9	−8.55 (−18.00, 0.90)	0.076	<0.001	87.9%	
**Baseline BMI (kg/m^2^)**						
Normal (18.5−24.9)	3	1.77 (−10.36, 13.92)	0.774	0.002	83.8%	0.177
Overweight (25−29.9)	9	−6.91 (−15.12, 1.30)	0.099	<0.001	89.6%	
Obese (>30)	2	−13.36 (−23.68, −3.03)	**0.011**	0.095	64.0%	
**Health status**						
Diabetic	7	−1.04 (−7.65, 5.55)	0.756	0.002	70.8%	0.044
Non-diabetic	16	−9.41 (−14.17, −4.65)	**<0.001**	<0.001	83.7%	
**Intervention**						
Saffron	12	−6.31 (−13.85, 1.21)	0.100	<0.001	88.9%	0.953
Crocin	9	−6.58 (−10.91, −2.25)	**0.003**	0.033	52.1%	
**Subgroup analyses of saffron on serum HDL (mg/dl)**						
Overall effect	23	0.21 (−0.73, 1.16)	0.660	<0.001	66.2%	
**Baseline HDL (mg/dl)**						
<40	4	−0.20 (−1.66, 1.25)	0.782	0.765	0.0%	0.668
≥40	18	0.22 (−1.07, 1.51)	0.738	<0.001	71.6%	
**Trial duration (week)**						
<12	15	0.07 (−1.14, 1.29)	0.902	<0.001	69.9%	0.726
≥12	8	0.43 (−1.17, 2.05)	0.595	0.015	59.9%	
**Intervention dose (mg/day)**						
<100	14	0.61 (−0.76, 2.00)	0.381	<0.001	76.1%	0.371
≥100	9	−0.15 (−1.13, 0.82)	0.755	0.371	7.8%	
**Baseline BMI (kg/m^2^)**						
Normal (18.5−24.9)	3	1.19 (−1.45, 3.84)	0.378	0.005	81.0%	0.682
Overweight (25−29.9)	9	−0.03 (−1.65, 1.59)	0.969	0.002	66.5%	
Obese (>30)	2	0.75 (−0.86, 2.36)	0.362	0.797	0.0%	
**Health status**						
Diabetic	7	0.14 (−1.29, 1.58)	0.843	0.018	60.8%	0.484
Non-diabetic	16	0.23 (−1.05, 1.52)	0.723	<0.001	69.3%	
**Intervention**						
Saffron	12	0.09 (−1.03, 1.22)	0.864	0.051	43.8%	0.669
Crocin	9	−0.33 (−1.93, 1.28)	0.688	<0.001	75.5%	
**Subgroup analyses of saffron on serum FBG (mg/dl)**						
Overall effect	22	−7.59 (−11.88, −3.30)	**0.001**	<0.001	93.3%	
**Baseline FBG (mg/dl)**						
<100	5	−6.55 (−12.14, −0.96)	**0.022**	<0.001	89.8%	0.510
≥100	16	−9.00 (13.70, −4.31)	**<0.001**	<0.001	66.1%	
**Trial duration (week)**						
<12	13	−4.77 (−9.91, 0.36)	0.068	<0.001	94.0%	0.079
≥12	9	−12.02 (−18.28, −5.77)	**<0.001**	<0.001	77.1%	
**Intervention dose (mg/day)**						
<100	16	−10.05 (−15.17, −4.92)	**<0.001**	<0.001	95.1%	0.018
≥100	6	−2.03 (−6.26, 2.20)	0.348	0.426	0.0%	
**Baseline BMI (kg/m^2^)**						
Normal (18.5−24.9)	3	−7.40 (−17.77, 2.97)	0.162	<0.001	95.6%	0.861
Overweight (25−29.9)	8	−8.59 (−16.57, −0.61)	**0.035**	0.004	66.4%	
Obese (>30)	4	5.57 (−13.03, 1.89)	0.144	0.070	57.6%	
**Health status**						
Diabetic	10	−14.08 (−22.38, −5.78)	**0.001**	<0.001	73.4%	0.047
Non-diabetic	12	−4.11 (−9.38, 1.15)	0.126	<0.001	95.8%	
**Intervention**						
Saffron	11	−7.49 (−13.98, −1.01)	**0.023**	<0.001	83.3%	0.897
Crocin	9	−8.13 (−15.41, −0.86)	**0.028**	<0.001	94.8%	
**Subgroup analyses of saffron on serum Insulin (mIU/ml)**						
Overall effect	7	−0.46 (−1.00, 0.06)	0.088	<0.001	75.6%	
**Trial duration (week)**						
<12	3	−0.50 (−1.43, 0.42)	0.285	0.005	81.5%	0.970
≥12	4	−0.53 (−1.39, 0.33)	0.229	0.004	77.7%	
**Intervention dose (mg/day)**						
<100	4	−0.96 (−2.10, 0.16)	0.094	<0.001	83.2%	0.121
≥100	3	−0.04 (−0.34, 0.26)	0.795	0.321	12.0%	
**Baseline BMI (kg/m^2^)**						
Overweight (25−29.9)	2	0.00 (−0.33, 0.33)	**0.002**	0.254	23.0%	0.008
Obese (>30)	2	−2.14 (−5.62, 1.33)	0.996	0.004	88.2%	
**Health status**						
Diabetic	4	−0.55 (−1.35, 0.24)	0.175	0.002	80.4%	0.836
Non-diabetic	3	−0.43 (−1.28, 0.41)	0.319	0.023	73.6%	
**Intervention**						
Saffron	3	−0.04 (−0.34, 0.26)	0.795	0.321	12.0%	0.146
Crocin	3	−1.41 (−3.23, 0.41)	0.130	<0.001	88.1%	
**Subgroup analyses of saffron on serum HbA1c (%)**						
Overall effect	12	−0.18 (−0.21, −0.07)	**<0.001**	0.008	56.9%	
**Trial duration (week)**						
<12	4	−0.11 (−0.24, 0.02)	0.104	0.088	54.1%	0.163
≥12	8	−0.27 (−0.45, −0.08)	**0.004**	0.008	63.1%	
**Intervention dose (mg/day)**						
<100	9	−0.21 (−0.33, −0.09)	**<0.001**	0.014	58.2%	0.050
≥100	3	−0.03 (−0.17, 0.09)	0.557	0.431	0.0%	
**Baseline BMI (kg/m^2^)**						
Overweight (25−29.9)	5	−0.14 (−0.25, −0.03)	**0.013**	0.178	36.5%	0.689
Obese (>30)	2	−0.36 (−0.94, 0.21)	0.214	0.088	65.7%	
**Health status**						
Diabetic	9	−0.25 (−0.46, −0.03)	**0.020**	0.003	65.1%	0.463
Non-diabetic	3	−0.17 (−0.22, −0.11)	**<0.001**	0.296	17.8%	
**Intervention**						
Saffron	7	−0.15 (−0.22, −0.08)	**<0.001**	0.342	11.5%	0.229
Crocin	4	−0.38 (−0.75, −0.01)	**0.042**	0.001	81.7%	
**Subgroup analyses of saffron on HOMA−IR**						
Overall effect	7	−0.49 (−0.89, −0.09)	**0.016**	0.002	70.8%	
**Trial duration (week)**						
<12	2	−0.23 (−0.41, −0.04)	**0.013**	0.618	0.0%	0.147
≥12	5	−1.19 (−2.49, 0.09)	0.070	<0.001	80.3%	
**Intervention dose (mg/day)**						
<100	5	−1.22 (−2.42, −0.02)	**0.045**	0.001	77.7%	0.088
≥100	2	−0.15 (−0.42, 0.10)	0.246	0.234	29.4%	
**Baseline BMI (kg/m^2^)**						
Overweight (25−29.9)	2	−1.03 (−4.62, 2.55)	0.071	0.174	45.9%	0.527
Obese (>30)	2	−1.14 (−2.91, 0.62)	0.573	<0.001	91.8%	
**Health status**						
Diabetic	4	−0.68 (−1.40, 0.04)	0.066	<0.001	83.6%	0.422
Non-diabetic	3	−0.32 (−0.79, 0.15)	0.180	0.326	10.8%	
**Intervention**						
Saffron	3	−0.17 (−0.49, 0.15)	0.305	0.206	36.8%	0.236
Crocin	3	−1.07 (−2.53, 0.38)	0.149	0.001	86.7%	
**Subgroup analyses of saffron on SBP (mmHg)**						
Overall effect	11	−3.42 (−5.80, −1.04)	**0.005**	<0.001	82.5%	
**Baseline SBP (mmHg)**						
<120	6	−2.83 (−6.29, 0.62)	0.108	<0.001	78.7%	0.602
≥120	5	−4.24 (−8.22, −0.25)	**0.037**	0.001	79.3%	
**Trial duration (week)**						
<12	6	−2.81 (−6.03, 0.41)	0.088	0.001	76.6%	0.601
≥12	5	−4.21 (−8.38, −0.05)	**0.047**	<0.001	83.0%	
**Intervention dose (mg/day)**						
<100	3	−4.97 (−8.06, −1.88)	**0.002**	0.114	53.9%	0.293
≥100	8	−2.67 (−5.64, 0.29)	0.078	<0.001	81.1%	
**Baseline BMI (kg/m^2^)**						
Overweight (25−29.9)	4	−4.88 (−9.93, 0.16)	0.058	<0.001	90.5%	0.501
Obese (>30)	3	−2.01 (−8.67, 4.64)	0.553	0.004	82.2%	
**Health status**						
Diabetic	4	−4.54 (−9.34, 0.26)	0.064	<0.001	83.8%	0.576
Non-diabetic	7	−2.91 (−5.99, 0.17)	0.064	0.001	74.4%	
**Intervention**						
Saffron	8	−2.67 (5.64, 0.29)	0.078	<0.001	81.1%	0.069
Crocin	2	−6.41 (−9.12, −3.69)	**<0.001**	0.446	0.0%	
**Subgroup analyses of saffron on DBP (mmHg)**						
Overall effect	9	−0.19 (−2.42, 2.03)	0.862	<0.001	81.4%	
**Baseline DBP (mmHg)**						
<80	5	2.23 (−0.32, 4.79)	0.087	0.070	53.8%	0.017
≥80	4	−2.95 (−6.35, 0.43)	0.088	<0.001	83.8%	
**Trial duration (week)**						
<12	6	−0.25 (−3.52, 3.02)	0.880	<0.001	87.7%	0.787
≥12	3	0.29 (−1.88, 2.46)	0.793	0.764	0.0%	
**Baseline BMI (kg/m^2^)**						
Overweight (25−29.9)	4	−2.02 (−5.44, 1.39)	0.246	<0.001	84.7%	0.706
Obese (>30)	3	−1.24 (−3.46, 0.98)	0.275	0.506	0.0%	
**Health status**						
Diabetic	4	−1.23 (−1.41, −1.05)	**<0.001**	0.485	0.0%	0.471
Non-diabetic	5	0.83 (−4.79, 6.47)	0.771	<0.001	89.9%	
**Subgroup analyses of saffron on serum CRP (mg/l)**						
Overall effect	10	−0.20 (−0.46, 0.05)	0.127	<0.001	74.4%	
**Trial duration (week)**						
<12	3	−0.22 (−0.84, 0.39)	0.478	<0.001	87.7%	0.805
≥12	7	−0.31 (−0.65, 0.03)	0.074	0.001	71.1%	
**Intervention dose (mg/day)**						
<100	5	−0.08 (−0.42, 0.24)	0.603	0.001	77.8%	0.061
≥100	5	−0.72 (−1.30, −0.14)	**0.014**	0.001	76.0%	
**Baseline BMI (kg/m^2^)**						
Normal (18.5−24.9)	2	−0.37 (−1.74, 0.99)	0.590	<0.001	93.9%	0.220
Overweight (25−29.9)	5	−0.40 (−0.94, 0.13)	0.144	0.004	71.2%	
**Health status**						
Diabetic	3	−0.05 (−0.24, 0.13)	0.572	0.466	0.0%	0.048
Non-diabetic	7	−0.52 (−0.94, −0.10)	**0.015**	<0.001	82.1%	
**Intervention**						
Saffron	6	−0.57 (−1.12, −0.02)	**0.040**	0.001	73.5%	0.327
Crocin	3	−0.19 (−0.72, 0.34)	0.489	<0.001	87.7%	
**Subgroup analyses of saffron on serum IL−6 (pg/ml)**						
Overall effect	3	−0.12 (−0.83, 0.59)	0.739	<0.001	87.4%	
**Subgroup analyses of saffron on serum TNF-α (pg/ml)**						
Overall effect	7	−2.54 (−4.43, −0.65)	**0.008**	<0.001	93.6%	
**Trial duration (week)**						
<12	2	−6.22 (−10.31, −2.14)	**0.003**	0.216	34.7%	0.009
≥12	5	−0.55 (−1.76, 0.66)	0.375	<0.001	78.1%	
**Intervention dose (mg/day)**						
<100	2	−2.84 (−7.45, 1.75)	0.226	<0.001	97.8%	0.704
≥100	5	−4.02 (−7.94, −0.10)	**0.044**	<0.001	80.9%	
**Baseline BMI (kg/m^2^)**						
Overweight (25−29.9)	4	−2.95 (−6.81, 0.89)	0.133	0.001	79.1%	0.797
Obese (>30)	2	−4.39 (−14.67, 5.88)	0.402	0.013	83.8%	
**Health status**						
Diabetic	3	−0.91 (−3.21, 1.37)	0.433	0.050	66.5%	0.103
Non-diabetic	4	−5.44 (−10.38, −0.51)	**0.031**	<0.001	95.4%	
**Subgroup analyses of saffron on Weight (Kg)**						
Overall effect	13	−0.12 (−0.82, 0.58)	0.732	0.995	0.0%	
**Trial duration (week)**						
<12	9	−0.07 (−0.82, 0.67)	0.840	0.960	0.0%	0.512
≥12	4	−0.75 (−2.62, 1.12)	0.431	0.959	0.0%	
**Intervention dose (mg/day)**						
<100	4	−0.16 (−1.20, 0.87)	0.757	0.695	0.0%	0.989
≥100	9	−0.17 (−1.10, 0.75)	0.714	0.989	0.0%	
**Health status**						
Diabetic	3	0.20 (−1.05, 1.45)	0.755	0.999	0.0%	0.488
Non-diabetic	10	−0.33 (−1.16, 0.50)	0.434	0.978	0.0%	
**Intervention**						
Saffron	9	0.02 (−0.73, 0.78)	0.954	0.996	0.0%	0.661
Crocin	2	−0.63 (−3.46, 2.19)	0.661	0.804	0.0%	
**Subgroup analyses of saffron on BMI (kg/m^2^)**						
Overall effect	12	0.01 (−0.17, 0.21)	0.853	0.809	0.0%	
**Trial duration (week)**						
<12	10	0.01 (−0.18, 0.20)	0.910	0.670	0.0%	0.574
≥12	2	0.27 (−0.62, 1.16)	0.548	0.981	0.0%	
**Intervention dose (mg/day)**						
<100	4	−0.18 (−0.57, 0.20)	0.346	0.428	0.0%	0.222
≥100	8	0.09 (−0.12, 0.31)	0.417	0.947	0.0%	
**Health status**						
Diabetic	3	0.12 (−0.12, 0.36)	0.338	0.999	0.0%	0.223
Non-diabetic	9	−0.12 (−0.42. 0.18)	0.428	0.783	0.0%	
**Intervention**						
Saffron	9	0.08 (−0.12, 0.28)	0.429	0.971	0.0%	0.456
Crocin	2	−0.23 (−1.03, 0.57)	0.569	0.714	0.0%	
**Subgroup analyses of saffron on WC (cm)**						
Overall effect	14	−1.50 (−2.83, −0.18)	**0.026**	<0.001	71.6%	
**Trial duration (week)**						
<12	8	−0.20 (−1.10, 0.70)	0.662	0.322	13.8%	0.110
≥12	6	−2.18 (−4.44, 0.07)	0.058	<0.001	77.2%	
**Intervention dose (mg/day)**						
<100	5	−2.68 (−4.88, −0.48)	**0.017**	0.018	66.3%	0.151
≥100	9	−0.70 (−2.25, 0.84)	0.370	<0.001	71.4%	
**Health status**						
Diabetic	3	−1.92 (−5.83, 1.98)	0.334	<0.001	87.7%	0.692
Non-diabetic	11	−1.09 (−2.32, 0.13)	0.080	0.006	58.0%	
**Intervention**						
Saffron	9	−0.80 (−2.33, 0.72)	0.304	0.001	68.6%	0.134
Crocin	2	−3.32 (−6.24, −0.40)	**0.026**	0.207	37.1%	
**Subgroup analyses of saffron on FM (%)**						
Overall effect	5	−0.57 (−1.57, 0.42)	0.262	0.599	0.0%	
**Trial duration (week)**						
<12	3	−0.42 (−1.44, 0.60)	0.422	0.762	0.0%	0.131
≥12	2	−3.05 (−6.31, 0.20)	0.066	0.726	0.0%	
**Intervention dose (mg/day)**						
<100	2	−0.82 (−2.35, 0.69)	0.287	0.845	0.0%	0.982
≥100	3	−0.85 (−2.57, 0.87)	0.332	0.341	10.4%	
**Subgroup analyses of saffron on MDA (uM/L)**						
Overall effect	7	−1.50 (−2.42, −0.57)	**0.001**	<0.001	77.4%	
**Trial duration (week)**						
<12	3	−2.08 (−4.17, −0.01)	0.050	<0.001	91.5%	0.304
≥12	4	−0.96 (−1.48, −0.43)	**<0.001**	0.537	0.0%	
**Intervention dose (mg/day)**						
<100	3	−1.32 (−3.10, 0.45)	0.145	<0.001	90.2%	0.881
≥100	4	−1.17 (−1.88, −0.47)	**0.001**	0.222	29.9%	
**Health status**						
Diabetic	2	−1.02 (−2.05, −0.01)	**0.049**	0.549	0.0%	0.484
Non-diabetic	5	−1.52 (−2.48, −0.57)	**0.002**	<0.001	80.9%	
**Intervention**						
Saffron	5	−1.08 (−1.69, −0.46)	**0.001**	0.306	16.7%	0.650
Crocin	2	−1.62 (−3.91, 0.66)	0.163	<0.001	95.1%	
**Subgroup analyses of saffron on TAC (mM/L)**						
Overall effect	7	0.07 (0.01, 0.13)	**0.032**	0.003	69.9%	
**Trial duration (week)**						
<12	3	0.04 (−0.01, 0.10)	0.121	0.035	70.2%	0.180
≥12	4	0.16 (−0.00, 0.33)	0.056	0.005	73.5%	
**Intervention dose (mg/day)**						
<100	3	0.03 (−0.01, 0.07)	0.132	0.091	58.2%	0.033
≥100	4	0.21 (0.05, 0.37)	**0.009**	0.033	61.8%	
**Health status**						
Diabetic	2	−0.01 (−0.13, 0.11)	0.836	0.645	0.0%	0.044
Non-diabetic	5	0.14 (0.05, 0.23)	**0.001**	<0.001	83.5%	
**Intervention**						
Saffron	5	0.17 (0.02, 0.31)	**0.021**	0.009	67.2%	0.087
Crocin	2	0.03 (−0.01, 0.08)	0.173	0.029	79.0%	
**Subgroup analyses of saffron on ALT (U/L)**						
Overall effect	11	−2.16 (−4.10, −0.23)	**0.028**	<0.001	88.8%	
**Trial duration (week)**						
<12	4	−5.58 (−10.42, −0.75)	**0.024**	<0.001	95.3%	0.036
≥12	7	−0.17 (−1.61, 1.26)	0.811	0.099	41.8%	
**Intervention dose (mg/day)**						
<100	8	−3.01 (−6.20, 0.19)	0.065	<0.001	91.6%	0.197
≥100	3	−0.71 (−2.09, 0.66)	0.310	0.285	20.9%	
**Health status**						
Diabetic	5	0.19 (−0.95, 1.34)	0.738	0.041	59.9%	0.003
Non-diabetic	6	−5.10 (−8.41, −1.78)	**0.003**	<0.001	78.0%	
**Intervention**						
Saffron	5	−0.05 (−1.68, 1.57)	0.944	0.112	44.1%	0.043
Crocin	5	−4.94 (−9.38, −0.50)	**0.029**	<0.001	94.6%	
**Subgroup analyses of saffron on AST(U/L)**						
Overall effect	11	1.03 (−1.85, 3.92)	0.482	<0.001	96.3%	
**Trial duration (week)**						
<12	4	0.86 (−5.49, 7.22)	0.789	<0.001	98.6%	0.995
≥12	7	0.88 (−1.95, 3.73)	0.541	<0.001	86.3%	
**Intervention dose (mg/day)**						
<100	8	1.40 (−2.82, 5.64)	0.514	<0.001	96.3%	0.338
≥100	3	−0.77 (−2.18, 0.64)	0.285	0.229	30.6%	
**Health status**						
Diabetic	5	−1.26 (−1.85, −0.66)	**<0.001**	0.349	10.0%	0.155
Non-diabetic	6	2.46 (−2.63, 7.56)	0.342	<0.001	93.2%	
**Intervention**						
Saffron	5	−0.05 (−1.82, 1.71)	0.950	0.094	46.8%	0.841
Crocin	5	−0.60 (−5.64, 4.43)	0.814	<0.001	97.5%	
**Subgroup analyses of saffron on ALP(U/L)**						
Overall effect	5	2.84 (−14.29, 19.97)	0.745	0.544	82.6%	
**Trial duration (week)**						
<12	2	−4.48 (−37.38, 28.42)	0.790	0.023	80.5%	0.510
≥12	3	7.75 (−7.86, 23.37)	0.330	0.146	48.1%	

ALP, alkaline phosphatase; ALT, alanine transaminase; AST, aspartate transaminase; BMI, body mass index; CI, confidence interval; CRP, c-reactive protein; FBG, fasting blood glucose; FM, fat mass; HbA1c, hemoglobin A1c; HDL, high-density lipoprotein; HOMA-IR, homeostatic model assessment for insulin resistance; LDL, low-density lipoprotein; DBP, diastolic blood pressure; MDA, malondialdehyde; SBP, systolic blood pressure; TAC, total antioxidant capacity; TC, total cholesterol, TG, triglyceride; WC, waist circumference; WMD, weighted mean differences; IL-6, interleukin 6.

Subgroup analyses have been done.

*P* < 0.05 was considered a significance. Bold means significant *p*-value (*P* < 0.05).

Different subgroup analyses were performed to determine the potential sources of heterogeneity among studies. The subgroup analysis revealed that saffron significantly decreased TG in studies with < 12 weeks of intervention (WMD = −11.18 mg/dl; 95%CI: −18.53, −3.84; P = 0.003), low (WMD = −7.80 mg/dl; 95%CI: −14.44, −1.16; P = 0.021) and high (WMD = −11.29 mg/dl; 95%CI: −20.69, −1.89; P = 0.019) doses of intervention, subjects with baseline TG < 150 (WMD = −4.65 mg/dl; 95%CI: −8.88, −0.43; P = 0.031), non-diabetic participants (WMD = −10.66 mg/dl; 95%CI: −17.70, −3.62; P = 0.003), and in studies that used saffron (WMD = −8.96 mg/dl; 95%CI: −16.01, −1.93; P = 0.013) as intervention, but when the baseline BMI was > 30 kg/m^2^, saffron significantly increased TG levels (WMD = 17.93 mg/dl; 95%CI: −35.31, −0.55; P = 0.043). Also, the reduction in TC and LDL levels was significant in some subgroups. In studies with ≥ 12 weeks intervention duration (WMD = −11.21 mg/dl; 95%CI: −19.74, −2.69; P = 0.010), intervention dose ≥ 100 mg/day (WMD = −10.76 mg/dl; 95%CI: −18.12, −3.41; P = 0.004), studies that used crocin (WMD = −7.15 mg/dl; 95%CI: −9.98, −4.33; P < 0.001)], obese (WMD = −19.59 mg/dl; 95%CI: −33.56, −5.61; P = 0.006) and normal weight (WMD = −7.43 mg/dl; 95%CI: −10.52, −4.34; P < 0.001) participants, non-diabetic individuals (WMD = −7.77 mg/dl; 95%CI: −13.03, −2.50; P = 0.004), and subjects with baseline TC < 200 (WMD = −7.39 mg/dl; 95%CI: −13.16, −1.62; P = 0.012), TC levels were reduced. The following subgroups showed a reduction in LDL: baseline LDL ≥ 100 (WMD = −8.10 mg/dl; 95%CI: −14.38, −1.82; P = 0.011), intervention dose < 100 mg/day (WMD = −5.10 mg/dl; 95%CI: −5.10, −1.23; P = 0.010), using crocin (WMD = −6.58 mg/dl; 95%CI: −10.91, −2.25; P = 0.003) as an intervention, obese (WMD = −13.36 mg/dl; 95%CI: −23.68, −3.03; P = 0.011) and non-diabetic (WMD = −9.41 mg/dl; 95%CI: −14.17, −4.65; P < 0.001) participants (Table 3).

#### Effect of saffron consumption on glycemic profiles and subgroup analysis

Saffron’s effects on FBG, insulin, HbA1c, and HOMA-IR were calculated in nineteen (22 effect sizes) (15, 17, 42, 44, 45, 47, 49, 51–54, 57, 58, 60–62, 64, 66, 67) with 1231 participants (IG = 616, CG = 615), six (7 effect sizes) (15, 44, 45, 57, 58, 64) with 422 participants (IG = 212, CG = 210), ten (12 effect sizes) with 761 participants (IG = 380, CG = 381) (15, 17, 44, 45, 54, 57, 60–62, 64) and six (7 effect sizes) (15, 45, 57, 58, 61, 64) trials with 405 participants (IG = 202, CG = 203), respectively. Pooled random-effects model analysis revealed significant decreasing effects of saffron on FBG level (WMD = −7.59 mg/dl, 95%CI: −11.88, −3.30; *P* = 0.001; *I*^2^ = 93.3%, *P* < 0.001; Figure 2E), HbA1c (WMD = −0.18%, 95%CI: −0.21, −0.07; *P* < 0.001; *I*^2^ = 56.9%, *P* = 0.008; Figure 2G), and HOMA-IR (WMD = −0.49, 95%CI: −0.89, −0.09; *P* = 0.016; *I*^2^ = 70.8%, *P* = 0.002; Figure 2H). However, the effects of saffron on serum insulin level (WMD = −0.46 miu/ml, 95%CI: −1.00, 0.06; *P* = 0.088; *I*^2^ = 75.6%, *P* < 0.001; Figure 2F) were not significant.

A subgroup analysis revealed that saffron at doses of less than 100 mg per day could considerably lower FBG levels (WMD = −10.05; 95%CI: −15.17, −4.92; *P* < 0.001), HbA1c (WMD = −0.21; 95%CI: −0.33, −0.09; *P* < 0.001) and HOMA-IR (WMD = −1.22; 95%CI: −2.42, −0.02; *P* = 0.045). The results also showed that saffron could significantly reduce FBG level and HbA1c, when the duration of intervention was ≥ 12 weeks (WMD _FBG_ = −12.02 mg/dl; 95%CI: −18.28, −5.77; *P* < 0.001; WMD _HbA1*c*_ = −0.27%; 95%CI: −0.45, −0.08; *P* = 0.004), and HOMA-IR, when the length of intervention was less than 12 weeks (WMD = −0.23; 95%CI: −0.41, −0.04; *P* = 0.013). Furthermore, both diabetic (WMD = −0.25%; 95%CI: −0.46, −0.03; *P* = 0.020) and non-diabetic (WMD = −0.17%; 95%CI: −0.22, −0.11; *P* < 0.001) participants who consumed saffron had significantly lower HbA1c levels. Saffron, however, only significantly affects FBG levels in diabetic patients (WMD = −14.08 mg/dl; 95%CI: −22.38, −5.78; *P* = 0.001). Additionally, the subgroup analysis showed that only the overweight patients’ serum insulin concentrations (WMD = −0.00 miu/ml; 95%CI: −0.33, 0.33; *P* = 0.002) could be considerably lowered by saffron.

Both saffron (WMD = −7.49 mg/dl; 95%CI: −13.98, −1.01; *P* = 0.023) and crocin (WMD = −8.13 mg/dl; 95%CI: −15.41, −0.86; *P* = 0.028) consumption resulted in significantly lower FBG levels, however, only saffron consumption resulted in significantly lower HbA1c (WMD = −0.15%; 95%CI: −0.22, 0.08; *P* < 0.001) values (Table 3).

#### Effect of saffron consumption on blood pressure and subgroup analysis

In total, we pooled data from 9 (11 effect sizes) (40, 45, 48, 52, 53, 57, 63, 64, 66), six (7 effect sizes) with 564 participants (IG = 285, CG = 279), and 8 (9 effect sizes) with 476 participants (IG = 241, CG = 235), (40, 48, 52, 53, 57, 63, 64, 66) studies to evaluate the effect of saffron on SBP and DBP, respectively. The pooled effect demonstrated a significant reduction in SBP after consuming saffron (WMD = −3.42 mmHg, 95%CI: −5.80, −1.04; *P* = 0.005; *I*^2^ = 82.5%, *P* < 0.001; Figure 2I). Saffron had not significant effect on DPB (WMD = −0.19 mmHg, 95%CI: −2.42, 2.03; *P* = 0.862; *I*^2^ = 81.4%, *P* < 0.001; Figure 2J). A subgroup analysis revealed that saffron at doses of <100 mg/day (WMD = −4.97 mmHg; 95%CI: −8.06, −1.88; *P* = 0.002) for ≥ 12 weeks (WMD = −4.21 mmHg; 95%CI: −8.38, −0.05; *P* = 0.047) in patients with baseline SBP ≥ 120 (WMD = −4.24 mmHg; 95%CI: −8.22, −0.25; *P* = 0.037), and when crocin (WMD = −6.41 mmHg; 95%CI: −9.12, −3.69; *P* < 0.001) was used as an intervention, could significantly lower SBP. The results also showed that saffron could significantly reduce DBP in diabetic patients (WMD = −1.23 mmHg; 95%CI: −1.41, −1.05; *P* < 0.001) (Table 3).

#### Effect of saffron consumption on inflammatory markers and subgroup analysis

Saffron’s effect on CRP, IL-6, and TNF-α was studied in 8 (10 effect sizes) (44, 46, 52, 57, 58, 62, 65, 68) with 596 participants (IG = 299, CG = 297), 3 (3 effect sizes) (53, 62, 66) with 165 participants (IG = 84, CG = 81), and 7 (7 effect sizes) studies with 427 participants (IG = 215, CG = 212), (53, 57, 59, 62, 65, 66, 68), respectively. A meta-analysis revealed that saffron significantly reduced TNF-α (WMD = −2.54 pg/ml, 95%CI: −4.43, −0.65; *P* = 0.008; *I*^2^ = 93.6%, *P* < 0.001; Figure 2M), and a subgroup analysis revealed that saffron had a significant influence on TNF-α in studies with < 12 weeks of intervention (WMD = −6.22 pg/ml; 95%CI: −10.31, −2.14; *P* = 0.003), and high dose interventions (≥ 100 mg/day) (WMD = −4.02 pg/ml; 95%CI: −7.94, −0.10; *P* = 0.044).

The variations in CRP (WMD = −0.20 mg/l, 95%CI: −0.46, 0.05; *P* = 0.127; *I*^2^ = 74.4%, *P* < 0.001; Figure 2K), and IL-6 (WMD = −0.12 pg/ml, 95%CI: −0.83, 0.59; *P* = 0.739; *I*^2^ = 87.4%, *P* < 0.001; Figure 2L) when compared to controls were not significant. Saffron consumption, on the other hand, resulted in significant decreases in CRP in high dose interventions (≥100 mg/day) (WMD = −0.72 mg/l; 95%CI: −1.30, −0.14; *P* = 0.014), non-diabetic subjects (WMD = −0.52 mg/l; 95%CI: −0.94, −0.10; *P* = 0.015) and when saffron (WMD = −0.57 mg/l; 95%CI: −1.12, −0.02; *P* = 0.040) used as intervention (Table 3).

#### Effect of saffron consumption on anthropometric parameters and subgroup analysis

Changes in body weight, BMI, WC, and FM% were assessed in 12 (13 effect sizes) (41, 44, 48–50, 52, 55, 57, 58, 60, 68, 69) with 841 participants (IG = 425, CG = 416), 11 (12 effect sizes) with 785 participants (IG = 396, CG = 389) (44, 48–50, 52, 57, 58, 60, 63, 68, 69), 15 (7 effect sizes) with 884 participants (IG = 447, CG = 437) (41, 44, 45, 48, 49, 52, 53, 55, 57, 60, 68, 69), and 4 (5 effect sizes) (41, 49, 57, 68) trials with 100 participants (IG = 50, CG = 50), respectively. Overall, we observed no significantly different change in weight (WMD = −0.12 kg, 95%CI: −0.82, 0.58; *P* = 0.732; *I*^2^ = 0.0%, *P* = 0.995; Figure 2N), BMI (WMD = 0.01 kg/m^2^, 95%CI: −0.17, 0.21; *P* = 0.853; *I*^2^ = 0.0%, *P* = 0.809; Figure 2O), and FM% (WMD = −0.57%, 95%CI: −1.57, 0.42; *P* = 0.262; *I*^2^ = 0.0%, *P* = 0.599; Figure 2Q) between the intervention and control groups. However, pooled effect sizes showed a substantial decrease in WC after saffron consumption (WMD = −1.50 cm; 95%CI: −2.83, −0.18; *P* = 0.026; *I*^2^ = 71.06%, *P* < 0.001; Figure 2P). A subgroup analysis revealed that saffron at doses of less than 100 mg per day (WMD = −2.68 cm; 95%CI: −4.88, −0.48; *P* = 0.017) could dramatically lower WC. Also, when crocin was used as an intervention, we saw a significant reduction in WC (WMD = −3.32 cm; 95%CI: −6.24, −0.40; *P* = 0.026) (Table 3).

#### Effect of saffron consumption on the immune system and subgroup analysis

For MDA and TAC, the study comprised and 455 subjects (IG: 230, CG: 225), and 454 subjects (IG:229, CG:225) from 7 trials (7 effect sizes) respectively (57–59, 62, 65, 68, 69), According to the meta-analysis, saffron had a decreasing effect on MDA (WMD = −1.50 uM/L, 95%CI: −2.42, −0.57; *P* = 0.001; *I*^2^ = 77.4%, *P* < 0.001; Figure 2R) and an enhancing effect on TAC (WMD = 0.07 mM/L, 95%CI: 0.01, 0.13; *P* = 0.032; *I*^2^ = 69.9%, *P* = 0.003; Figure 2S). The subgroup analysis revealed that MDA in both diabetic (WMD = −1.02 uM/L; 95%CI: −2.05, −0.01; *P* = 0.049) and non-diabetic (WMD = −1.52 uM/L; 95%CI: −2.48, −0.57; *P* = 0.002) patients decreased significantly after consuming saffron. Saffron also significantly raised TAC in non-diabetic subjects (WMD = 0.14 mM/L; 95%CI: 0.05, 0.23; *P* = 0.001), according to subgroup analysis. In studies which used saffron as an intervention (WMD _MDA_ = −1.08 uM/L; 95%CI: −1.69, −0.46; *P* = 0.001; WMD _TAC_ = 0.17 uM/L; 95%CI: 0.02, 0.31; *P* = 0.021), and studies with intervention doses of ≥100 (WMD _MDA_ = −1.17 uM/L; 95%CI: −1.88, −0.47; *P* = 0.001; WMD _TAC_ = 0.21 mM/L; 95%CI: 0.05, 0.37; *P* = 0.009) saffron significantly reduced MDA while increasing TAC. In studies with interventions lasting more than 12 weeks (WMD = −0.96 uM/L; 95%CI: −1.48, −0.43; *P* < 0.001), saffron dramatically decreased MDA, according to additional subgroup analyses (Table 3).

#### Effect of saffron consumption on liver enzymes and subgroup analysis

Saffron significantly affected ALT (WMD = −2.16 U/L, 95%CI: −4.10, −0.23; *P* = 0.028; *I*^2^ = 88.8%, *P* < 0.001; Figure 2T), according to the findings of a pooled analysis of 8 studies (11 effect sizes) (15, 42, 46, 54, 57, 61, 67, 68) with 637 participants (IG = 318, CG = 319). However, the results of a pooled analysis of 8 (12 effect sizes) (15, 42, 46, 54, 57, 61, 67, 68) with 637 participants (IG = 318, CG = 319) and 4 (5 effect sizes) (42, 46, 57, 61) trials with 296 participants (IG = 148, CG = 148), revealed no significant effect of saffron on AST (WMD = 1.03 U/L, 95%CI: −1.85, 3.92; *P* = 0.482; *I*^2^ = 96.3%, *P* < 0.001; Figure 2U) and ALP (WMD = 2.84 U/L, 95%CI: −14.29, 19.97; *P* = 0.745; *I*^2^ = 82.6%, *P* = 0.544; Figure 2V) respectively. The subgroup analysis revealed that saffron results in 5.58 (U/L) and 5.10 (U/L) reductions in ALT compared to controls in studies with a duration < 12 weeks (WMD = −5.58 U/L; 95%CI: −10.42, −0.75; *P* = 0.024) and non-diabetic patients (WMD = −5.10 U/L; 95%CI: −8.41, −1.78; *P* = 0.003), respectively. Crocin (WMD = −4.94 U/L; 95%CI: −9.38, −0.50; *P* = 0.029), when taken as an intervention, could dramatically lower AST. Additionally, after consuming saffron, the overweight individuals’ AST levels (WMD = −1.26 U/L; 95%CI: −1.85, −0.66; *P* < 0.001) significantly decreased (Table 3).

### Non-linear dose-response analysis

There was evidence of a non-linear relationship between saffron dosage and HDL (coefficients = 5.95, *P* = 0.049; Figure 4D), HOMA-IR (coefficients = 7.69, *P* = 0.002; Figure 4H), weight (coefficients = 0.06, *P* = 0.036; Figure 4N), and ALP (coefficients = 1.78, *P* = 0.016; Figure 4V). In addition, the non-linear dose-response analysis revealed a non-linear relationship between saffron dosage and FBG (coefficients = −0.67, *P* = 0.011; Figure 4E), HbA1c (coefficients = −0.02, *P* = 0.002; Figure 4G), and TNF-α (coefficients = −3.56, *P* = 0.042; Figure 4M).

**FIGURE 3:**
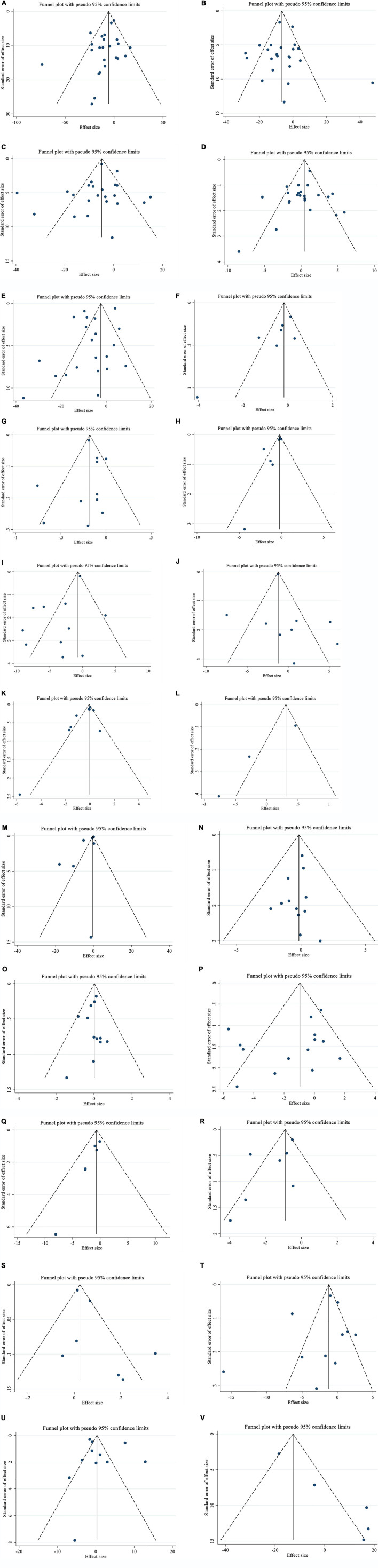
Funnel plots for the effect of saffron consumption on **(A)** TG (mg/dl); **(B)** TC (mg/dl); **(C)** LDL (mg/dl); **(D)** HDL (mg/dl); **(E)** FBG (mg/dl); **(F)** Insulin (miu/ml); **(G)** HbA1c (%); **(H)** HOMA-IR; **(I)** SBP (mmHg); **(J)** DBP (mmHg); **(K)** CRP (mg/l); **(L)**; IL-6 (pg/ml); **(M)** TNF-α (pg/ml); **(N)** weight (kg); **(O)** BMI (kg/m^2^); **(P)** WC (cm); **(Q)** FM (%); **(R)** MDA (uM/L); **(S)** TAC (mM/L); **(T)** ALT (U/L); **(U)** AST (U/L) and **(V)** ALP (U/L). TG, triglyceride; TC, total cholesterol; LDL, low-density lipoprotein; HDL, high-density lipoprotein; FBG, fasting blood glucose; HOMA-IR, homeostasis model assessment for insulin resistance; HbA1c, hemoglobin A1c; CRP, C-reactive protein; IL-6, interleukin 6; TNF-α, tumor necrosis factor; TAC, total antioxidant capacity; BMI, body mass index; WC, waist circumference; FM, fat mass; ALT, alanine transaminase; AST, aspartate transaminase; ALP, alkaline phosphatase; MDA, malondialdehyde*;* SBP, systolic blood pressure; DBP, diastolic blood pressure; CI, confidence interval; WMD, weighted mean difference.

**FIGURE 4:**
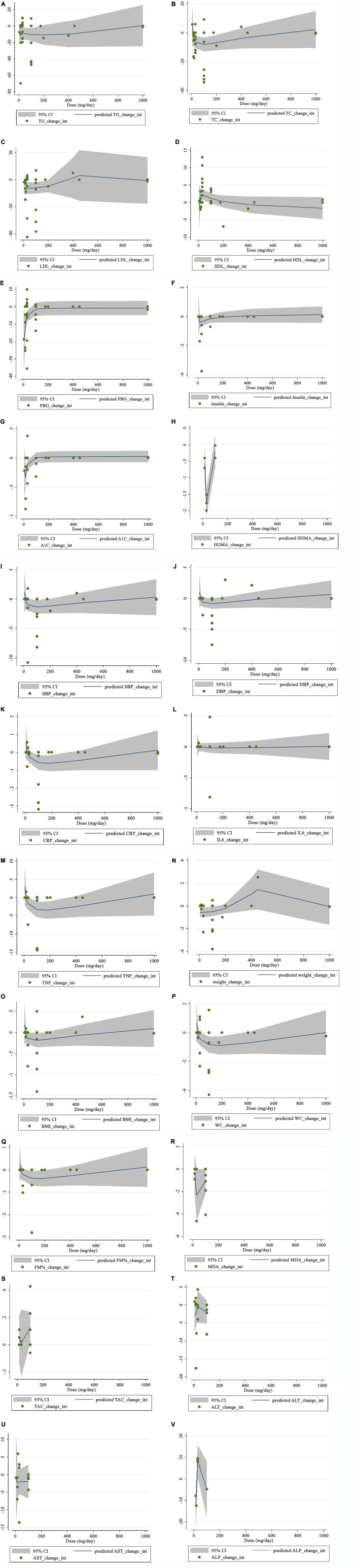
Non-linear dose-response relations between saffron consumption and absolute mean differences. Dose-response relations between dose (mg/day) and absolute mean differences **(A)** TG (mg/dl); **(B)** TC (mg/dl); **(C)** LDL (mg/dl); **(D)** HDL (mg/dl); **(E)** FBG (mg/dl); **(F)** Insulin (miu/ml); **(G)** HbA1c (%); **(H)** HOMA-IR; **(I)** SBP (mmHg); **(J)** DBP (mmHg); **(K)** CRP (mg/l); **(L)**; IL-6 (pg/ml); **(M)** TNF-α (pg/ml); **(N)** weight (kg); **(O)** BMI (kg/m^2^); **(P)** WC (cm); **(Q)** FM (%); **(R)** MDA (uM/L); **(S)** TAC (mM/L); **(T)** ALT (U/L); **(U)** AST (U/L) and **(V)** ALP (U/L). TG, triglyceride; TC, total cholesterol; LDL, low-density lipoprotein; HDL, high-density lipoprotein; FBG, fasting blood glucose; HOMA-IR, homeostasis model assessment for insulin resistance; HbA1c, hemoglobin A1c; CRP, C-reactive protein; IL-6, interleukin 6; TNF-α, tumor necrosis factor; TAC, total antioxidant capacity; BMI, body mass index; WC, waist circumference; FM, fat mass; ALT, alanine transaminase; AST, aspartate transaminase; ALP, alkaline phosphatase; MDA, malondialdehyde*;* SBP, systolic blood pressure; DBP, diastolic blood pressure; CI, confidence interval; WMD, weighted mean difference.

**FIGURE 5:**
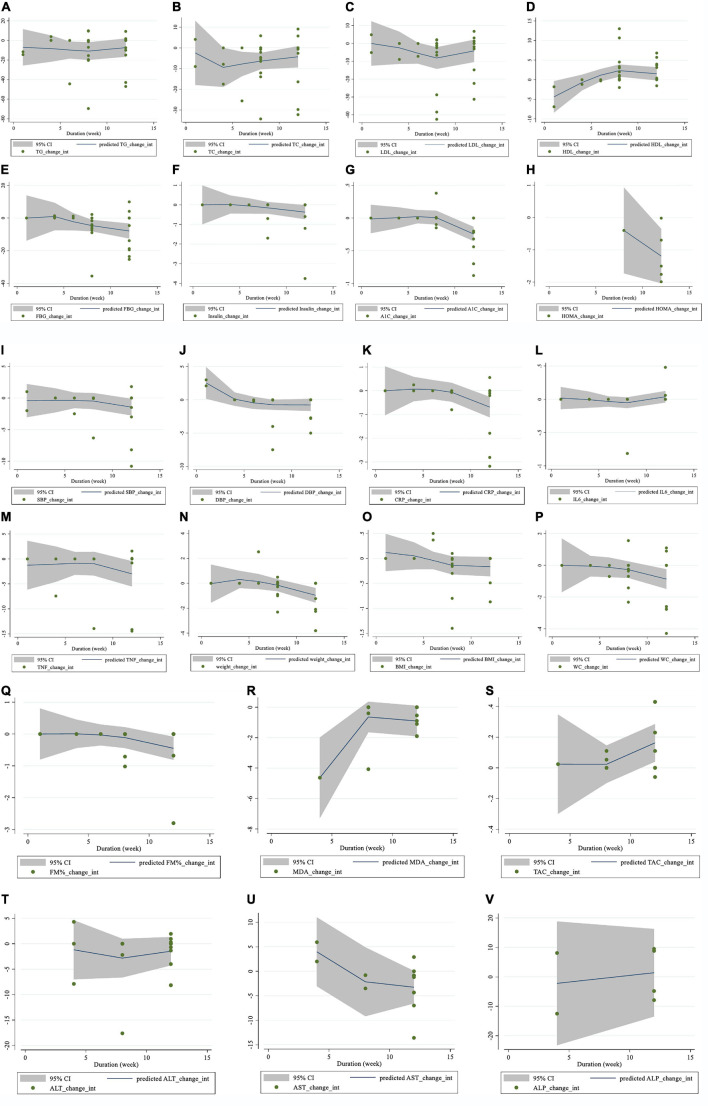
Non-linear dose-response relations between saffron consumption and absolute mean differences. Dose-response relations between duration of intervention (week) and absolute mean differences on **(A)** TG (mg/dl); **(B)** TC (mg/dl); **(C)** LDL (mg/dl); **(D)** HDL (mg/dl); **(E)** FBG (mg/dl); **(F)** Insulin (miu/ml); **(G)** HbA1c (%); **(H)** HOMA-IR; **(I)** SBP (mmHg); **(J)** DBP (mmHg); **(K)** CRP (mg/l); **(L)**; IL-6 (pg/ml); **(M)** TNF-α (pg/ml); **(N)** weight (kg); **(O)** BMI (kg/m^2^); **(P)** WC (cm); **(Q)** FM (%); **(R)** MDA (uM/L); **(S)** TAC (mM/L); **(T)** ALT (U/L); **(U)** AST (U/L) and **(V)** ALP (U/L). TG, triglyceride; TC, total cholesterol; LDL, low-density lipoprotein; HDL, high-density lipoprotein; FBG, fasting blood glucose; HOMA-IR, homeostasis model assessment for insulin resistance; HbA1C, hemoglobin A1C; CRP, C-reactive protein; IL-6, interleukin 6; TNF-α, tumor necrosis factor; TAC, total antioxidant capacity; BMI, body mass index; WC, waist circumference; FM, fat mass; ALT, alanine transaminase; AST, aspartate transaminase; ALP, alkaline phosphatase; MDA, malondialdehyde*;* SBP, systolic blood pressure; DBP, diastolic blood pressure; CI, confidence interval; WMD, weighted mean difference.

Moreover, there was a non-linear relationship between the length of the intervention and HDL (coefficients = 3.20, *P* = 0.007; Figure 5D) and DBP (coefficients = −1.85, *P* = 0.033; Figure 5J). However, there was no evidence of a non-linear association between the duration of the intervention and other outcomes.

### Meta-regression analysis

**FIGURE 6:**
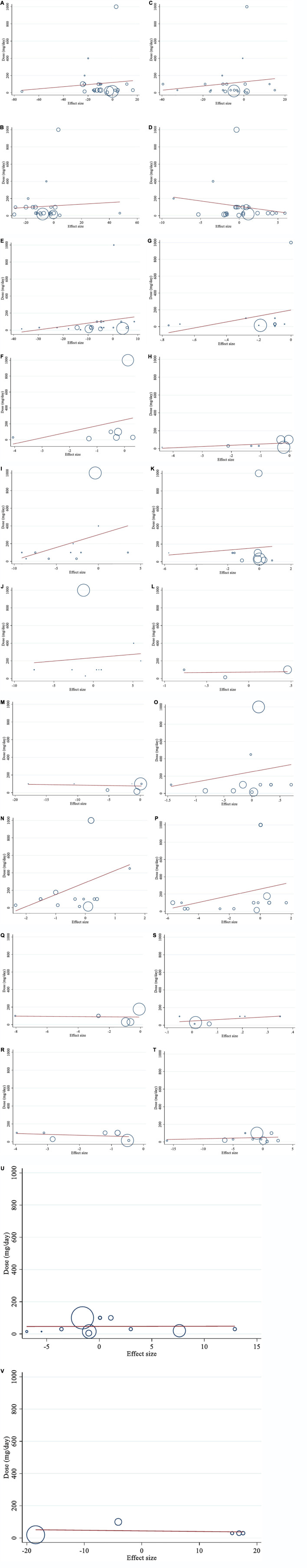
Linear dose-response relations between saffron consumption and absolute mean differences. Dose-response relations between dose (g/day) and absolute mean differences **(A)** TG (mg/dl); **(B)** TC (mg/dl); **(C)** LDL (mg/dl); **(D)** HDL (mg/dl); **(E)** FBG (mg/dl); **(F)** Insulin (miu/ml); **(G)** HbA1c (%); **(H)** HOMA-IR; **(I)** SBP (mmHg); **(J)** DBP (mmHg); **(K)** CRP (mg/l); **(L)**; IL-6 (pg/ml); **(M)** TNF-α (pg/ml); **(N)** weight (kg); **(O)** BMI (kg/m^2^); **(P)** WC (cm); **(Q)** FM (%); **(R)** MDA (uM/L); **(S)** TAC (mM/L); **(T)** ALT (U/L); **(U)** AST (U/L) and **(V)** ALP (U/L). TG, triglyceride; TC, total cholesterol; LDL, low-density lipoprotein; HDL, high-density lipoprotein; FBG, fasting blood glucose; HOMA-IR, homeostasis model assessment for insulin resistance; HbA1C, hemoglobin A1C; CRP, C-reactive protein; IL-6, interleukin 6; TNF-α, tumor necrosis factor; TAC, total antioxidant capacity; BMI, body mass index; WC, waist circumference; FM, fat mass; ALT, alanine transaminase; AST, aspartate transaminase; ALP, alkaline phosphatase; MDA, malondialdehyde*;* SBP, systolic blood pressure; DBP, diastolic blood pressure; CI, confidence interval; WMD, weighted mean difference.

**FIGURE 7:**
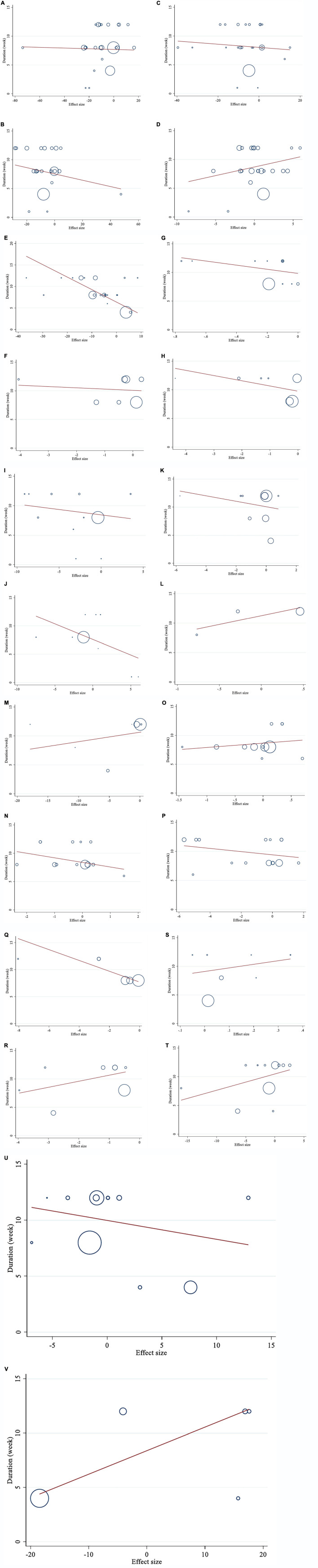
Linear dose-response relations between saffron consumption and absolute mean differences. Dose-response relations between duration of intervention (week) and absolute mean differences **(A)** TG (mg/dl); **(B)** TC (mg/dl); **(C)** LDL (mg/dl); **(D)** HDL (mg/dl); **(E)** FBG (mg/dl); **(F)** Insulin (miu/ml); **(G)** HbA1c (%); **(H)** HOMA-IR; **(I)** SBP (mmHg); **(J)** DBP (mmHg); **(K)** CRP (mg/l); **(L)**; IL-6 (pg/ml); **(M)** TNF-α (pg/ml); **(N)** weight (kg); **(O)** BMI (kg/m^2^); **(P)** WC (cm); **(Q)** FM (%); **(R)** MDA (uM/L); **(S)** TAC (mM/L); **(T)** ALT (U/L); **(U)** AST (U/L) and **(V)** ALP (U/L). TG, triglyceride; TC, total cholesterol; LDL, low-density lipoprotein; HDL, high-density lipoprotein; FBG, fasting blood glucose; HOMA-IR, homeostasis model assessment for insulin resistance; HbA1C, hemoglobin A1C; CRP, C-reactive protein; IL-6, interleukin 6; TNF-α, tumor necrosis factor; TAC, total antioxidant capacity; BMI, body mass index; WC, waist circumference; FM, fat mass; ALT, alanine transaminase; AST, aspartate transaminase; ALP, alkaline phosphatase; MDA, malondialdehyde*;* SBP, systolic blood pressure; DBP, diastolic blood pressure; CI, confidence interval; WMD, weighted mean difference.

Meta-regression analysis was used to assess how the dosage of saffron and the length of the intervention altered lipid profiles, glycemic profiles, blood pressure, inflammatory markers, anthropometric parameters, the immune system, and liver enzymes. Linear association was found between FBG and duration of intervention (coefficients = −0.29, P = 0.003; Figure 7E). There was no statistically significant linear association between the length and dosage of the intervention and changes in other outcomes (Figures 6 A–V, 7 A–D, F–V).

### Sensitivity analysis

Findings regarding saffron consumption and lipid profiles, blood pressure, FBG, HbA1c, HOMA-IR, IL-6, weight, BMI, FM%, MDA, TAC, AST, and ALP remained robust in the sensitivity analysis. However, the significant effect of saffron on TNF-α, WC, and ALT disappeared when excluding the studies done by Ghiasian et al. (59) (WMD = −0.86, 95%CI: −2.19, 0.46) and Hamidi et al. (65) (WMD = −1.72, 95%CI: −3.45, 0.01) for TNF-α; Fadai et al. (A) (45) (WMD = −1.11, 95%CI: −2.36, 0.13), Fadai et al. (B) (45) (WMD = −1.08, 95%CI: −2.31, 0.14), Abedimanesh et al. (B) (49) (WMD = −1.29, CI 95%: −2.58, 0.01), Kermani et al. (52) (WMD = −1.18, 95%CI: −2.44, 0.07), and Ebrahimi et al. (57) (WMD = −0.87, 95%CI: −1.90, 0.16) for WC; Mohamadpour et al. (43) (WMD = −1.51, 95%CI: −3.20, 0.16), Parsi et al. (67) (WMD = −1.24, 95%CI: −2.83, 0.35), and Tajaddini et al. (15) (WMD = −2.52, 95%CI: −5.11, 0.06) for ALP. Sensitivity analysis indicated that exclusion of the articles done by Mohamadpour et al. (43) (WMD = −0.36, 95%CI: −0.65, −0.06), Azimi et al. (44) (WMD = −0.33, 95%CI: −0.66, −0.01), Mousavi et al. (A) (46) (WMD = −0.38, 95%CI: −0.74, −0.02), Mousavi et al. (B) (46) (WMD = −0.33, 95%CI: −0.66, −0.00), Ebrahimi et al. (57) (WMD = −0.33, 95%CI: −0.66, −0.00), and Shahbazian et al. (62) (WMD = −0.29, 95%CI: −0.57, −0.02) altered the overall effect of saffron on CRP concentration to a significant value. Additionally, the total effect of saffron on insulin was significantly changed by excluding the study by Fadai et al. (A) (45) (WMD = −0.61, 95%CI: -1.21, -0.01).

### GRADE assessment

The GRADE system is used to grade the quality of the evidence by the outcome in Table 4. For TG, TC, LDL, FBG, HbA1c, HOMA-IR, SBP, weight, WC, MDA, TAC, and ALT, the quality of the evidence was very low. Additionally, the HDL, DBP, CRP, IL-6, TNF-, BMI, FM%, and AST evidence quality was low. Only for insulin and ALP were the evidence quality levels moderate and high, respectively.

**TABLE 4:** GRADE profile of saffron on CVD risk factors in adults.

Outcomes	Risk of bias	Inconsistency	Indirectness	Imprecision	Publication bias	WMD (95%CI)	Quality of evidence
TG	No serious limitation	serious limitation ^1^	No serious limitation	No serious limitation	No serious limitation	−8.81 (−14.33, −3.28)	⊕◯◯◯ Very low
TC	No serious limitation	serious limitation ^1^	No serious limitation	No serious limitation	No serious limitation	−6.87 (−11.19, −2.56)	⊕◯◯◯ Very low
LDL	No serious limitation	Very serious limitation ^1^	No serious limitation	No serious limitation	No serious limitation	−6.71 (−10.51, −2.91)	⊕◯◯◯ Very low
HDL	No serious limitation	serious limitation ^1^	No serious limitation	Serious limitation^2^	No serious limitation	0.21 (−0.73, 1.16)	⊕⊕◯◯ Low
FBG	No serious limitation	Very serious limitation ^1^	No serious limitation	No serious limitation	No serious limitation	−7.59 (−11.88, −3.30)	⊕◯◯◯ Very low
Insulin	No serious limitation	Very serious limitation ^1^	No serious limitation	Serious limitation^2^	serious limitation	−0.46 (−1.00, 0.06)	⊕⊕⊕◯ Moderate
HbA1c	No serious limitation	serious limitation ^1^	No serious limitation	No serious limitation	No serious limitation	−0.18 (−0.21, −0.07)	⊕◯◯◯ Very low
HOMA−IR	No serious limitation	serious limitation ^1^	No serious limitation	No serious limitation	No serious limitation	−0.49 (−0.89, −0.09)	⊕◯◯◯ Very low
SBP	No serious limitation	Very serious limitation ^1^	No serious limitation	No serious limitation	No serious limitation	−3.42 (−5.80, −1.04)	⊕◯◯◯ Very low
DBP	No serious limitation	Very serious limitation ^1^	No serious limitation	Serious limitation^2^	No serious limitation	−0.19 (−2.42, 2.03)	⊕⊕◯◯ Low
CRP	No serious limitation	Very serious limitation ^1^	No serious limitation	Serious limitation^2^	No serious limitation	−0.20 (−0.46, 0.05)	⊕⊕◯◯ Low
IL-6	No serious limitation	Very serious limitation ^1^	No serious limitation	Serious limitation^2^	No serious limitation	−0.12 (−0.83, 0.59)	⊕⊕◯◯ Low
TNF-α	No serious limitation	Very serious limitation ^1^	No serious limitation	No serious limitation	No serious limitation	−2.54 (−4.43, −0.65)	⊕⊕◯◯ Low
Weight	No serious limitation	No serious limitation	No serious limitation	Serious limitation^2^	No serious limitation	−0.12 (−0.82, 0.58)	⊕◯◯◯ Very low
BMI	No serious limitation	No serious limitation	No serious limitation	Serious limitation^2^	No serious limitation	0.01 (−0.17, 0.21)	⊕⊕◯◯ Low
WC	No serious limitation	serious limitation ^1^	No serious limitation	No serious limitation	No serious limitation	−1.50 (−2.83, −0.18)	⊕◯◯◯ Very low
FM	No serious limitation	No serious limitation	No serious limitation	Serious limitation^2^	serious limitation	−0.57 (−1.57, 0.42)	⊕⊕◯◯ Low
MDA	No serious limitation	serious limitation ^1^	No serious limitation	No serious limitation	No serious limitation	−1.50 (−2.42, −0.57)	⊕◯◯◯ Very low
TAC	No serious limitation	Very serious limitation ^1^	No serious limitation	No serious limitation	No serious limitation	0.07 (0.01, 0.13)	⊕◯◯◯ Very low
ALT	No serious limitation	Very serious limitation ^1^	No serious limitation	No serious limitation	No serious limitation	−2.16 (−4.10, −0.23)	⊕◯◯◯ Very low
AST	No serious limitation	Very serious limitation ^1^	No serious limitation	Serious limitation^2^	No serious limitation	1.03 (−1.85, 3.92)	⊕⊕◯◯ Low
ALP	serious limitation	Very serious limitation ^1^	No serious limitation	Serious limitation^2^	serious limitation	2.84 (−14.29, 19.97)	⊕⊕⊕⊕ High

^1^ALP, alkaline phosphatase; ALT, alanine transaminase; AST, aspartate transaminase; BMI, body mass index; CI, confidence interval; CRP, c-reactive protein; FBG, fasting blood glucose; FM, fat mass; HbA1c, hemoglobin A1c; HDL, high-density lipoprotein; HOMA-IR, homeostatic model assessment for insulin resistance; LDL, low-density lipoprotein; DBP, diastolic blood pressure; MDA, malondialdehyde; SBP, systolic blood pressure; TAC, total antioxidant capacity; TC, total cholesterol, TG, triglyceride; WC, waist circumference; IL-6, interleukin 6.

^2^There is significant heterogeneity for TG (*I*^2^ = 55.1%), TC (*I*^2^ = 72.5%), LDL (*I*^2^ = 81.3%), HDL (*I*^2^ = 66.2%), FBG (*I*^2^ = 93.3%), Insulin (*I*^2^ = 75.6%), HbA1C (*I*^2^ = 56.9%), HOMA-IR (*I*^2^ = 70.8%), SBP (*I*^2^ = 82.5%), DBP (*I*^2^ = 81.4%), CRP (75.4%), IL-6 (*I*^2^ = 87.4%), TNF-α (*I*^2^ = 92.5%), WC (*I*^2^ = 70.3%), MDA (*I*^2^ = 73.7%), TAC (*I*^2^ = 77.3%), ALT (*I*^2^ = 87.7%), and AST (*I*^2^ = 95.9%) and ALP (*I*^2^ = 82.6.9%).

There is no evidence of significant effects of saffron consumption on HDL, Insulin, DBP, CRP, IL-6, Weight, BMI, FM, AST, and ALP.

### Publication bias

There was no evidence of publication bias among the included articles assessing the effect of saffron on TG (*P*_Egger_ = 0.077, *P*_Begg_ = 0.413; Figure 3A), TC (P_Egger_ = 0.950, *P*_Begg_ = 0.916; Figure 3B), LDL (*P*_Egger_ = 0.410, *P*_Begg_ = 0.958; Figure 3C), HDL (*P*_Egger_ = 0.352, *P*_Begg_ = 0.958; Figure 3D), FBG (*P*_Egger_ = 0.0.074, P_Begg_ = 1.00; Figure 3E), HbA1c (*P*_Egger_ = 0.866, *P*_Begg_ = 0.273; Figure 3G), HOMA-IR (*P*_Egger_ = 0.059, *P*_Begg_ = 0.133; Figure 3H), DBP (*P*_Egger_ = 0.529, *P*_Begg_ = 0.348; Figure 3J), IL-6 (P_*Egger*_ = 0.108, *P*_Begg_ = 1.00; Figure 3L), TNF-α (*P*_Egger_ = 0.130, *P*_Begg_ = 1.00; Figure 3M), weight (*P*_Egger_ = 0.183, *P*_Begg_ = 0.702; Figure 3N), BMI (P_Egger_ = 0.382, *P*_Begg_ = 0.542; Figure 3O), WC (*P*_Egger_ = 0.238, *P*_Begg_ = 0.216; Figure 3P), MDA (*P*_Egger_ = 0.105, *P*_Begg_ = 0.138; Figure 3R), TAC (*P*_Egger_ = 0.050, P_Begg_ = 0.621; Figure 3S), ALT (*P*_*Egger*_ = 0.403, *P*_Begg_ = 0.131; Figure 3T), and AST (*P*_Egger_ = 0.829, *P*_Begg_ = 784; Figure 3U) levels, using Begg’s test and Egger’s tests. But among articles evaluating the impact of saffron on insulin (*P*_Egger_ = 0.041, *P*_Begg_ = 0.072; Figure 3F), SBP (*P*_Egger_ = 0.042, *P*_Begg_ = 1.00; Figure 3I), CRP (P_Egger_ = 0.023, *P*_Begg_ = 0.697; Figure 3K) FM% (*P*_Egger_ = 0.001, *P*_Begg_ = 0.060; Figure 3Q), and ALP (*P*_Egger_ = 0.004, *P*_Begg_ = 0.462; Figure 3V), publication biases were found.

## Discussion

The present study is a comprehensive systematic review and dose-response meta-analysis of the effects of saffron on all CVD risk factors. The results of 32 RCT with 1674 individuals showed that saffron intake can reduce TG, TC, LDL, FBG, HbA1c, HOMA-IR, SBP, CRP, TNF-α, WC, MDA, and ALT, and can elevate TAC levels. According to the subgroup analysis TG, TC, and LDL were reduced significantly in individuals with obesity, and FBG was reduced in overweight individuals. Moreover, participants with diabetes showed a significant reduction in FBG, HBA1c, and MDA levels by saffron supplementation. Saffron supplementation reduced LDL and SBP in individuals with abnormal baseline levels (LDL ≥ 100 mg/dl and SBP ≥ 120 mmHg), and reduced TG and TC in the categories of lower levels (TG < 150 mg/dl and TC < 200 mg/dl). This supplementation also reduced FBG in both categories of baseline higher and lower than 100 mg/dl. In the non-linear dose-response analysis, between dose for saffron intake and HDL, HOMA-IR, ALP, HbA1c, TNF-α, FBG, and weight was a significant association, and a significant linear association was seen between FBG and duration of saffron supplementation.

Saffron (crocus sativus) is a nutraceutical containing three phytochemical compounds including carotenoids (crocin and crocetin) that are responsible for saffron color, volatile oil component (safranal) that produces odor, and glycoside (picrocrocin) that is the bitter precursor for safranal (70–72). These different subtypes have different tastes, odors, absorption ways, and bioavailability (21). When the hydrophobic crocetin is esterified with two water-soluble sugars (gentiobioses), crocin will be produced which is water soluble and has a high bioavailability. The included studies in this meta-analysis have used two types of substances (saffron or crocin) for supplementation. According to the subgroup analysis, TG, CRP, MDA, and TAC were reduced only in the saffron group while LDL SBP, WC, and ALT were reduced in the crocin group. Both of these compounds could effectively reduce TC, FBG, and HbA1c.

### The effect of saffron on inflammatory markers

This study revealed reductions in CRP and TNF-α but no changes in IL-6 were seen following the saffron intervention. A meta-analysis of 8 RCTs in 2021 by Asbaghi et al. did not reveal any significant impacts of saffron on CRP, TNF-α, and IL-6. However, significant reductions occurred in subgroups with higher baseline measures (CRP ≥ 3 mg/l and TNF-α ≥ 15 pg/ml), lower supplementation dosages (≤30 mg/day), and some other subgroups (26). The controversy can be due to the larger sample size of the present study. The limited number of included trials evaluating the effect of saffron on IL-6 (only three studies) hindered the implementation of subgroup analyses on IL-6. In the present study, the subgroups of non-diabetic individuals and intervention dosages of more than 100 mg/d showed significantly lower CRP and TNF-α levels. Moreover, there was a non-linear association between dose with TNF-α.

Saffron can inhibit serum levels of inflammatory markers such as nuclear factor kappa B (NF-kB), TNF-α, Interferon-gamma (IFN-γ), and some interleukins while acting as the agonist of peroxisome proliferator-activated receptor γ (PPARγ) (73). This medical spice can also downregulate key pro-inflammatory enzymes such as myeloperoxidase (MPO), inducible nitric oxide synthase (iNOS), cyclooxygenase-2 (COX-2), phospholipase A2, and prostanoids (73).

### The effect of saffron on antioxidant status

Saffron could reduce MDA levels, and enhance TAC according to our analysis. MDA and TAC reduced significantly in the subgroups of intervention dose of ≥100 mg/d and MDA reduced in the subgroup of trial duration ≥12 months. Oxidative stress, which means the loss of balance between oxidants and antioxidants in favor of oxidants, occurs when the environmental stressors become overwhelming or in case of not enough antioxidant capacity in the body (74). A meta-analysis by Morvaridzadeh et al in 2021 showed the beneficial effect of saffron on TAC and MDA in unhealthy patients (20).

The mechanism by which saffron can affect oxidative stress can be attributed to increasing the levels of glutathione reductase (75). Safranal is suggested to act against the aging process due to its antioxidant properties and can act as a remedy for hepatic ischemia-reperfusion (IR) injury via the prevention of high intracellular reactive oxygen species (ROS) concentration and restoring the content of antioxidant enzymes (76). Existing research shows that saffron can enhance some antioxidant enzymes such as catalase and superoxide dismutase (SOD) (77). Moreover, animal studies revealed the anti-toxicity effects of saffron in different tissues against natural or chemical toxins (78).

### The effect of saffron on lipid profiles

The finding of this study shows significant reductions in TG, TC, and LDL after saffron supplementation. However, no significant changes in HDL levels were seen. A meta-analysis of six RCTs by Asbaghi et al. in 2019 demonstrated similar results in the reduction of TG and TC but showed a significant increase in HDL and no changes in LDL levels which are in contrast with this study (25). Another dose-response meta-analysis of 14 RCTs in 2019 by Rahmani et al. showed results similar to the Asbaghi et al. study in TC and TG reduction, no changes in LDL levels, and an increase in HDL levels after long-term consumption of saffron according to the meta-regression analysis (79). The optimum dose of saffron supplementation was 400 mg/d for TG reduction in this study, while dose-response analysis in the present study was not significant for TG (79). The controversy between these two studies in 2019 and the present study in 2022 can be related to the higher sample size of the present study owing to the recently published RCTs (15, 67). Moreover, another meta-analysis of ten studies also published in 2019 by Pourmasoumi et al. showed no effect of saffron on lipid profile (22). According to the subgroup analysis, the reduction in TC, TG, and LDL was significant in individuals with BMI ≥ 30 (obese). This can be explained by the anti-inflammatory properties of saffron (75) since inflammatory markers are higher in individuals with obesity compared to normal weight (80). A non-linear association has been seen between HDL levels with both the dose and duration of saffron intervention.

An animal study on the effect of saffron (crocetin) against alcoholic fatty liver showed that this substance can enhance mitochondrial-β-oxidation, decline fatty sediment, and prevent lipid peroxidation (19). Moreover, saffron and crocin could effectively reduce hyperlipidemia parameters in rats (81) and humans (79). Saffron also reduces cholesteryl ester transfer protein (CETP) which is involved in the regulation of serum lipid profile (51).

### The effect of saffron on insulin resistance

In this study saffron significantly impacted FBG, HbA1c, and HOMA-IR while not affecting insulin levels. A meta-analysis of ten studies in 2019 by Pourmasoumi et al. showed a significant reduction in fasting plasma glucose (FPG) following saffron supplementation and no changes in fasting insulin level (22). Another meta-analysis by Rahmani et al. in 2020 also showed a reduction in FPG especially when the intervention duration exceeds 12 weeks, but could not show a reduction in HbA1c (23). In contrast, another meta-analysis of six RCTs by Asbaghi et al. in 2019 revealed no significant changes in FBG after the supplementation (25). This is in line with the meta-analysis by Roshanravan et al. in 2022 that could not reveal any impact of saffron on blood glucose (82). The existing controversy can be due to different sample sizes, different participant morbidities, or different types of supplementations. According to the subgroup analysis FBG and HbA1c reduced significantly in individuals with diabetes after the saffron intervention. This can be justified by the anti-inflammatory properties of saffron in individuals with diabetes (83). There was shown a non-linear association between FBG, HbA1c, and HOMA-IR with a dose of saffron supplementation.

Regarding the effect of saffron on blood glucose profile, this agent enhances glucose uptake and insulin sensitivity in muscle cells via the phosphorylation of AMPK (AMP-activated protein kinase), ACC (acetyl-CoA carboxylase), and MAPKs (mitogen-activated protein kinase) (18).

### The effect of saffron on blood pressure

Our results showed a significant reduction in SBP and a non-significant reduction in DBP. In contrast, a meta-analysis of ten publications by Pourmasoumi et al. in 2019 showed a significant reduction in DBP and no changes in SBP following saffron supplementation. Another dose-response meta-analysis by Setayesh et al. in 2021 showed the effective impact of saffron on both SBP and DBP and mentioned that the impact of the supplementation on DBP is dependent on the duration of the intervention and the effect would be more in case of longer durations (84). Subgroup analysis of the present study showed that SBP reduces in individuals with baseline SBP ≥ 120, intervention duration ≥ 12, and intervention dose of <100. DBP significantly decreased in the subgroup of diabetic patients. The dose-response analysis revealed a significant association in DBP in the optimum duration of 2 weeks.

The effect of saffron on endothelial nitric oxide (NO) synthases can lead to the elevation of NO production and the lowering of blood pressure (85). Moreover, Crocetin can down-regulate intracellular adhesion molecule-1 (ICAM-1) protein expression (86). This effect can affect the renin-angiotensin system and lead to hypertension suppression (87).

### The effect of saffron on anthropometric measures

According to this meta-analysis, saffron intake can significantly reduce WC but has no significant effect on weight, BMI, and FM. However, the non-linear dose-response analysis showed that the optimum dose of 450 mg/d of the intervention can reduce weight. A dose-response meta-analysis of 14 studies by Rahmani et al. in 2019 could not show any significant effect of this intervention on weight (79). This result can be interpreted as the intervention dose being lower than optimum in this study. The mean dose of saffron administered in the meta-analysis by Rahmani et al. was 160 mg/d (79). An animal study by Mashmoul et al. in 2014 compared the anti-obesity effect of crocin and saffron. After inducing obesity in rats with a high-fat diet for 12 weeks, the supplementation showed a beneficial effect of saffron on prospective food consumption and LDL/HDL ratio while crocin had a beneficial effect on lipid profile (TG, and TC), and lowered the rate of body weight gain (88). This is in line with the present study in which WC was reduced only in the crocin subgroup but not in the saffron group. The justification can be related to the higher antioxidant properties of crocin compared to the same weight of saffron since crocin is the carotenoid component responsible for the color of saffron (72).

The medical herb of saffron can regulate the expression of leptin and adiponectin in adipose tissue (89) and inhibit the secretion of pancreatic and gastric lipase that regulates fat absorption (90). This effect can reduce central adipose tissue accumulation and decrease blood circulating leptin levels, leading to higher satiety perception (90).

### The effect of saffron on liver enzymes

The liver enzymes ALT, AST, and ALP were assessed. Only ALT reduces after saffron supplementation according to this meta-analysis. However, the dose-response analysis showed that at the optimum dose of 20 mg/d ALP can be reduced. A meta-analysis of 12 RCTs by Karimi et al in 2021 showed no beneficial effect of saffron on the mentioned three liver function markers (91). Another meta-analysis of nine RCTs in 2022 by Mousavi et al. showed results similar to this analysis on AST, ALT, and ALP. However, the dose-response analysis did not show any relationships (92). The existing controversy can be due to different sample sizes or different supplementation types (crocin or saffron) in these studies.

Liver enzymes may rise above normal levels in healthy individuals or stay normal in liver diseases (93). Regarding this unstable nature of liver enzymes, the results of this study on the effect of saffron on ALT, ASP, and ALP should be interpreted carefully. Moreover, existing diseases can affect liver enzyme levels differently (93) and the participants of this meta-analysis had different morbidities.

This study is the first comprehensive systematic review and dose-response meta-analysis on the effect of saffron on all cardiovascular risk factors. The strengths of this study are the use of the risk of bias assessment, GRADE classification of quality of evidence, non-linear dose-response analysis, subgroup analysis, sensitivity analysis, and meta-regression analysis that enhance the accuracy of the results. Moreover, the adverse effects reported in the study were summarized. The studies were included based on inclusion criteria with a variety of participants which provided the possibility of subgroup analysis and also can make the results eligible to be generalized. The randomized placebo-controlled design of included studies and the double- or triple-blind design of most of them are other strengths. However, some limitations also exist. The contrasting findings may be due to different supplementation types of saffron (crocin, crocetin, safranal, and picrocrocin). Although all studies used randomization, information on allocation concealment, randomization efficiency, and withdrawal was not consistently reported. The included studies were significantly heterogeneous. Some of the current meta-analysis outcomes were secondary outcomes in RCTs. Moreover, regarding the considerable number of the included studies, the types of measurements for outcomes could be different. The intra-assay coefficient of variation and inter-assay variability for biochemical kits in different studies might lead to heterogeneous results. Most of the studies were conducted in Iran due to the use of this plant as a spice in cooked foods, and therefore it seems that it cannot be generalized to other countries. Similarly, the anthropometric indices were measured by different scales and differently trained persons in the included studies. In addition, the blood pressure had been taken in different positions (seated or standing posture, supine position) which is another limitation. It is suggested that combining saffron with starchy food can enhance its bioavailability (21). Therefore, different timing of supplementation in the included RCTs, whether it was consumed simultaneously with food or not, could lead to different results. Another point to be mentioned is the high risk of bias in some of the included trials, highlighting the need for more high-quality clinical trials in the future.

## Conclusion

This systematic review and dose-response meta-analysis revealed the beneficial effects of saffron on cardiovascular risk factors including TG, TC, LDL, FBG, HbA1c, HOMA-IR, SBP, CRP, TNF-α, WC, MDA, TAC, and ALT. The non-linear dose-response analysis showed a significant association between dose for saffron intake with HDL, HOMA-IR, ALP, HbA1c, TNF-α, FBG, weight, and showed between the supplementation duration and HDL level, and DBP. Given the significant beneficial results, saffron seems to be an appropriate supplement and adjunct therapy along with other conventional medicine used for preventing or alleviating CVD risk factors.

## Data availability statement

The original contributions presented in the study are included in the article, further inquiries can be directed to the corresponding author/s.

## Author contributions

MoZ designed the study. MoZ and OA developed the search strategy. MaZ, MN, and MN-S extracted the data and conducted the analyses. MoZ, FG, and AH drafted the manuscript. MoZ, MN-S, and OA assessed the risk of bias of the meta-analyses. FS and OA interpreted the results and revised the manuscript. All authors read and approved the final manuscript.

## Abbreviations

CVD: cardiovascular disease
TG: triglyceride
TC: total cholesterol
LDL: low-density lipoprotein
HDL: high-density lipoprotein
FBG: fasting blood glucose
HOMA-IR: homeostasis model assessment for insulin resistance
HbA1c: hemoglobin A1c
CRP: C-reactive protein
IL-6: interleukin 6
TNF-α: tumor necrosis factor
TAC: total antioxidant capacity
BMI: body mass index
WC: waist circumference
FM: fat mass
ALT: alanine transaminase
AST: aspartate transaminase
ALP: alkaline phosphatase
MDA: malondialdehyde
SBP: systolic blood pressure
DBP: diastolic blood pressure
CI: confidence interval
WMD: weighted mean difference
MPO: myeloperoxidase
iNOS: inducible nitric oxide synthase
COX-2: cyclooxygenase-2
NF-kB: nuclear factor kappa B
IFN-γ: Interferon gamma
PPARγ: peroxisome proliferator-activated receptorγ
IR: ischemia-reperfusion
ROS: reactive oxygen species
SOD: super oxide dismutase
CETP: cholesteryl ester transfer protein
FPG: fasting plasma glucose
AMPK: AMP-activated protein kinase
ACC: acetyl-CoA carboxylase
MAPKs: mitogen-activated protein kinase
NO: nitric oxide
ICAM-1: intracellular adhesion molecule-1
GRADE: grading of recommendations assessment, development, and evaluation.
